# High-dose rifampicin and clarithromycin for 4 weeks versus standard-dose rifampicin and clarithromycin for 8 weeks to treat Buruli ulcer: An open-label, individually randomized, controlled trial in Ghana

**DOI:** 10.1371/journal.pntd.0014783

**Published:** 2026-09-29

**Authors:** Abigail Agbanyo, Yaw Ampem Amoako, Charles Opondo, Michael Ntiamoah Oppong, Adwoa Asante-Poku, Jacob Novignon, Joseph Tuffour, Ishaque Saim Mintah, Ruth Dede Tuwor, Dzifa Kofi Ahiatrogah, Richard Adjei Akuffo, Miriam Gborglah, Iris Mosweu, Catherine Pitt, Elizabeth Allen, Stephen L. Walker, Dorothy Yeboah-Manu, Michael Marks, Richard Odame Phillips, SHARP Collaboration

**Affiliations:** 1 Centre for Collaborative Research, Kwame Nkrumah University of Science and Technology, Kumasi, Ghana; 2 School of Medical Sciences, Kwame Nkrumah University of Science and Technology, Kumasi, Ghana; 3 Department of Medical Statistics and the Clinical Trials Unit, Faculty of Epidemiology and Population Health, London School of Hygiene and Tropical Medicine, London, United Kingdom; 4 Noguchi Memorial Institute for Medical Research, University of Ghana, Accra, Ghana; 5 Centre for Social Policy Studies, University of Ghana, Accra, Ghana; 6 Department of Global Health and Development, Faculty of Public Health and Policy, London School of Hygiene and Tropical Medicine, London, United Kingdom; 7 Department of Clinical Research, Faculty of Infectious and Tropical Diseases, London School of Hygiene and Tropical Medicine, London, United Kingdom; 8 Hospital for Tropical Diseases, University College Hospital, London, United Kingdom; Johns Hopkins University, UNITED STATES OF AMERICA

## Abstract

**Background:**

The current 8-week regimen of rifampicin and clarithromycin for Buruli ulcer (BU) is suboptimal. High-dose rifampicin could shorten treatment and improve outcomes. We evaluated whether a 4-week high-dose regimen could improve time to clearance of viable *Mycobacterium ulcerans*.

**Methodology:**

In this open-label, individually randomised trial conducted in Ghana, participants with PCR-confirmed BU were assigned (1:1) to either high-dose oral rifampicin (20mg/kg) with clarithromycin (15mg/kg) for 4 weeks (HR) or standard-dose rifampicin (10mg/kg) with clarithromycin (15mg/kg) for 8 weeks (SR). All wounds were dressed with DACC-coated dressings. The primary outcome was time to clearance of viable *M. ulcerans* assessed by 16S rRNA qPCR up to week 20.

**Findings:**

Between 26^th^ November 2021 and 31^st^ July 2024, 42 participants were randomised (HR:23, SR:19), which was short of the target (n = 112). The mean time to clearance was 4.9 weeks (SE 1.4) in the HR arm versus 7.0 weeks (SE 1.9) in the SR arm, with an adjusted mean difference of -0.5 weeks (95%CI -5.0 to 4.1, p = 0.835). No recurrences occurred in either arm. Paradoxical reactions were observed in 0/21 participants in the HR arm vs. 5/15 in the SR arm (adjusted OR 0.19, 95%CI 0.00-1.35, p = 0.102). Secondary infection rates were similar between groups.

**Conclusion:**

High‑dose rifampicin combination for 4 weeks was well tolerated in this population. A nominally lower proportion of paradoxical reactions was observed in the high-dose rifampicin arm compared with standard therapy. The trial did not find evidence of significantly shorter time to microbiological clearance or healing compared with standard therapy. Larger trials are needed to determine efficacy and safety.

**Trial Registration:**

Pan African Clinical Trials Repository**,** trial registration number: PACTR202011867644311 (https://pactr.samrc.ac.za/TrialDisplay.aspx?TrialID=14534).

## Author summary

Buruli ulcer (BU) is a neglected tropical disease that causes destructive skin lesions and long-term disability, particularly in rural communities. The current standard treatment, eight weeks of rifampicin and clarithromycin, is effective but lengthy, which can reduce adherence and increase the burden on patients and health services. This study explored whether a high-dose rifampicin regimen could offer a safe, shorter, and more convenient alternative. In a randomized clinical trial conducted in Ghana, we compared a 4-week high-dose rifampicin regimen with the standard 8-week treatment. We assessed how quickly the bacteria causing BU were cleared from the wounds of affected individuals and monitored safety outcomes. The results showed that the shorter, high-dose regimen was not superior but was well tolerated and achieved similar bacterial clearance and healing times compared to standard treatment although we enrolled fewer participants than planned and the intervention and control groups were not evenly balanced. Notably, patients receiving the high-dose regimen experienced fewer paradoxical reactions, a common inflammatory complication during treatment. These findings suggest that high-dose rifampicin may offer a promising strategy to shorten BU treatment time. However, larger studies are needed to confirm its efficacy, safety and potential to shorten treatment duration while maintaining good outcomes.

## Introduction

Buruli ulcer (BU) is a neglected tropical disease (NTD) caused by *Mycobacterium ulcerans* (*M. ulcerans*). While the mode of transmission remains unknown, BU is endemic in over 30 countries globally, with the highest burden in rural West Africa, including Ghana. Children under 15 years are most commonly affected, though individuals of any age may develop the disease [1].

Initial lesions present as painless subcutaneous nodules or dermal plaques, which enlarge over days to weeks and eventually ulcerate. Without treatment, ulcers can expand to cover entire limbs or the trunk, causing extensive disfigurement. Patients typically remain systemically well, but secondary bacterial infections may occur. Access to health facilities in rural regions is often limited, and advanced disease is common due to fear, stigma, skepticism about allopathic medicine, and the economic burden on poor families [2–4]. The World Health Organization (WHO) NTD roadmap aims to control BU by 2030, and a key priority is the development of shorter, more cost-effective treatments [5].

Over the past two decades, antibiotic therapy has improved healing and reduced recurrence rates to below 2%. However, the current WHO‑recommended regimen comprising oral rifampicin (10 mg/kg) plus clarithromycin for 8 weeks [6] remains suboptimal. Many affected individuals must travel long distances to complete treatment, and healing times vary considerably, even among patients with similar lesions and irrespective of the regimen used [7–10]. Approximately two-thirds of patients heal within 25 weeks of treatment initiation while the remaining one-third may take up to a year to heal [7,9]. Additional challenges with the current rifampicin-clarithromycin regimen are the relatively long 8-week duration, adverse effects and drug interactions especially in older populations.

Rifampicin, the backbone of BU treatment [11] is a potent inducer of drug-metabolizing enzymes and can reduce the effectiveness of various medicines, creating management challenges in some patients. The current 10 mg/kg dose is derived from tuberculosis (TB) guidelines. Emerging evidence indicates that higher‑dose rifampicin is safe and well tolerated [12,13], and several large‑scale TB trials are evaluating this strategy [14,15]. In a study using high dose rifampin among individuals with tuberculous meningitis, no evidence of survival benefit from high-dose rifampin was observed. However, drug-induced liver injury occurred in 8.0% of the participants in the high-dose group and 4.4% of those in the standard-dose group, but no deaths from drug-induced liver injury occurred [15]. Preliminary data in BU suggest that higher doses may accelerate clearance of viable *M. ulcerans* enabling regimen shortening [16]. Some data suggest that standard rifampicin/clarithromycin-containing regimens may result in suboptimal antibiotic exposures, particularly among patients with higher body weight, potentially contributing to treatment failure [17]. These findings highlight the importance of considering drug exposure as a determinant of treatment response and support further evaluation of dose optimization strategies. Collectively, these data suggest that high-dose rifampicin is likely to be a safe and potentially more efficacious approach for BU, but it has not yet been evaluated in a clinical trial.

A complementary strategy to address both high mycobacterial load and secondary wound infections is the use of dialkylcarbamoyl chloride (DACC)-coated dressings. These dressings irreversibly bind bacteria on wound surfaces, removing them when changed, thereby reducing bacterial load without antimicrobials. In other clinical contexts, DACC dressings have been shown to reduce bacterial load more effectively than silver‑impregnated dressings [18], and to reduce wound size reduction [19]. Compared with vaseline gauze, the standard wound dressing in most of West Africa, DACC dressings may offer superior outcomes.

High‑dose rifampicin (20 mg/kg) with DACC dressings could accelerate clearance of viable mycobacteria, reduce the risk of paradoxical reactions and secondary infections, and improve overall patient outcomes compared with standard‑dose rifampicin (10 mg/kg) plus DACC. Paradoxical reactions are inflammatory responses that can cause significant pain, swelling, and wound deterioration, often hindering the healing process and requiring additional clinical management [20]. A shorter, more effective regimen would also be more convenient for affected individuals, potentially improving adherence, reducing costs for affected persons, their families and healthcare systems, and ultimately contributing to WHO’s 2030 BU control targets. The aim of this study was to determine whether high‑dose oral rifampicin (20 mg/kg) combined with DACC dressings improves the time to clearance of viable mycobacteria from BU wounds compared with standard‑dose oral rifampicin (10 mg/kg) combined with DACC dressings.

## Methods

### Ethics statement

This was an open-label phase 3 prospective, randomised controlled trial. The trial was sponsored by the London School of Hygiene and Tropical Medicine (LSHTM) and executed in collaboration with the Kwame Nkrumah University of Science and Technology (KNUST) and the Noguchi Memorial Institute of Medical Research (NMIMR). The trial protocol [21] was approved by the Ghana Health Service Ethics Review Committee (GHS-ERC: 003/01/21), KNUST (CHRPE/AP/030/21), LSHTM (reference number, 22912), and the FDA [FDA/CT/2110 and FDA/CT/2110 (ext 1)] The study was registered on the Pan African Clinical Trials Repository with trial registration number PACTR202011867644311 (https://pactr.samrc.ac.za/TrialDisplay.aspx?TrialID=14534).

Written informed consent was obtained from all participants. Written parental or guardian consent was obtained for all under 18 and assent for those aged 13–17 years. A Data and Safety Monitoring Board (DSMB) operating independently of the trial was constituted to oversee the conduct of the trial. The DSMB reviewed the trial protocol and plan for analysis at the start of the trial and annually throughout the recruitment period to conduct its oversight role.

### Participants

Participants were recruited from 26^th^ November 2021–25^th^ January 2024 from two treatment centres in Ghana with extensive expertise in BU care: Pakro Health Centre and Wassa Akropong Municipal Hospital. These facilities are located in districts endemic for BU. Eligible participants were those presenting with a Buruli nodule, plaque, or ulcer, with or without associated oedema, aged between 5 and 80 years old, for whom a sample collected from their lesion was positive for *M. ulcerans* DNA by PCR for the IS*2404* repetitive genetic element. Children younger than 5 years were excluded primarily for safety reasons, as high-dose rifampicin was not part of the standard treatment regimen at the time of the study. In addition, the distinct pharmacokinetic characteristics and smaller body size of very young children may necessitate age-specific dosing formulations. Furthermore, the assessment of subjective outcomes, such as pain, and the maintenance of reliable follow-up and treatment adherence may be more challenging in this age group. Individuals were also excluded if pregnant or if either rifampicin or clarithromycin was contraindicated. Complete eligibility criteria are provided in the published trial protocol (S1 File) [21].

### Randomisation, allocation and treatment

Participants enrolled in the study were randomly individually assigned in a 1:1 ratio to one of the two treatment groups. A computer-generated random allocation sequence was prepared at the LSHTM using block randomization, stratified by site, BU Category (Category I-III), and specific randomization numbers placed in sealed, opaque envelopes. This process was conducted securely, with access restricted to prevent any possibility of viewing the allocation sequence, and was maintained by the study coordinator.

The control regimen was the WHO–recommended standard combination of rifampicin (10mg/kg) and clarithromycin (15mg/kg) given daily for 8 weeks. The intervention group was given rifampicin (20mg/kg) and clarithromycin (15mg/kg) daily for 4 weeks. Participants in both groups had their ulcers dressed with DACC-coated dressing and changed every 48 hours until the lesion healed. Adherence to trial medication was monitored through directly observed therapy supervised in the clinic and at home by a domiciliary treatment monitor. A time allowance was in place for missed doses. Community-based surveillance volunteers (CBSVs) within study districts supported participant follow-up and adherence to trial medications and dressings. Additional details are provided in the published protocol [21].

### Trial procedures

Every participant attended 15 scheduled review visits at Weeks 2, 4, 6, 8, and monthly thereafter until reaching 52 weeks after starting antibiotic treatment. Participants were monitored for at least 20 weeks, and any visits beyond this period were part of standard care practices. Participants samples were collected according to the assessment schedule (S1 Table).

At enrolment, we collected demographic data. At enrolment and follow-up, clinical, cost, health-related quality of life data, and photographs of lesions were collected. Data on pain assessment were recorded using a Visual Analogue Scale (VAS). Wound diameter (including the region of induration) and area were recorded by digital photography and measured by computer planimetry using a Silhouette Wound Imaging System (ARANZ, Wellington, New Zealand) at each scheduled clinical visit.

Swabs or fine needle aspirate (FNA) samples were processed for PCR IS*2404* using previously published methods in the laboratories that currently provide PCR confirmation for routine BU cases at Kumasi Centre for Collaborative Research in Tropical Medicine (KCCR) and NMIMR [22]. Samples were collected before and during treatment to assess the persistence of viable mycobacteria. Swabs were taken from the undermined edges of ulcerated lesions, or FNA samples from the centre of non-ulcerative lesions (nodule, plaque, and oedema) at weeks 2, 4, 8, 12, 16 and 20 (if lesions were not healed) for the combined *M. ulcerans* 16S rRNA reverse transcriptase/IS*2404* real-time qPCR [23].

For the combined 16S rRNA reverse transcriptase/IS*2404* real-time qPCR assay to assess viability of *M. ulcerans,* swabs and FNAs were stabilized in 500µl of RNA protect (Qiagen) and subjected to combined DNA/RNA extraction by the All-Prep DNA/RNA extraction kit (Qiagen). RNA was reverse-transcribed to cDNA. DNA and cDNA were subjected to IS*2404* qPCR and 16S rRNA real time PCR respectively [23]. The median turn-around time for IS2404 qPCR assay was 5 days as previously published [24].

Wounds were assessed clinically for secondary infection at each time point. In the context of a new purulent discharge, a single swab was taken for microbiological assessment at KCCR and NMIMR Laboratories using previously published methods [25,26].

Transportation cost of participants between their homes and the health facilities was reimbursed.

Blinding was not possible for clinical research staff administering drugs. A skilled wound assessor blinded to the allocation evaluated wound images for assessment of healing to avoid bias and maximize the validity of results. Clinical and laboratory data were collected by staff blinded to the study arm.

### Outcomes

The primary outcome was the time to clearance of viable mycobacteria, evaluated up to week 20. The time to clearance was based on the duration from randomisation (which was same day on which antibiotic treatment was started) until the participant had no viable organisms, as assessed by the combined 16S rRNA reverse transcriptase/IS*2404* real-time qPCR assay. Participants with healed wounds or no evidence of viable mycobacteria by week 20 were considered to have had a favourable outcome.

Secondary outcomes included the proportion of participants achieving complete healing at 20 weeks, the proportion of participants with recurrence, the incidence of paradoxical reaction, and the incidence of secondary infection. Incidence of recurrences, paradoxical reaction, and secondary infection were assessed at 12 months following randomization. Definition of terms

Complete healing was defined as re-epithelialization of the ulcerated area with stable scar formation.

Recurrence was defined as the appearance of a new Buruli ulcer lesion within 12 months of completing antibiotic treatment, after complete healing of the initial lesion, and not considered a paradoxical reaction. This was assessed by the presence of viable micro-organisms detected by *M. ulcerans* culture or the combined *M. ulcerans* IS2404-16S rRNA assay.

Paradoxical reactions were defined as:

- an initial improvement in the clinical appearance of lesion upon antimicrobial treatment of BU followed by deterioration of the lesion or its surrounding tissues
- or the appearance of a new lesion(s)
- or examination of excised tissue from the clinical lesion showing evidence of an intense inflammatory reaction consistent with a paradoxical reaction.

Secondary infections were evaluated by trained clinicians using a standardized checklist that assessed the presence of pain, erythema, warmth, swelling, and purulent discharge to identify signs suggesting secondary bacterial infection as previously described [27]. A secondary infection was defined as the occurrence of new purulent discharge within a BU lesion in the presence of clinical signs of infection and accompanied by microbiological evidence of infection.

### Statistical methods

It was estimated that 50 participants in each arm would be required to have 80% power to detect a 2-week reduction in mean time to *M. ulcerans* clearance using statistical testing at the 5% level of significance. This estimate was based on previous studies showing a mean time to clearance of viable mycobacterium with standard dose oral rifampicin of 9 weeks (3 weeks standard deviation) [23].This sample size would also be sufficient to detect a 27% absolute increase in the proportion of participants with healed wounds by 20 weeks, assuming 50% would heal at 20 weeks in the standard dose arm. Allowing for a 10% rate of loss-to-follow-up, the study aimed to recruit a total of 112 participants.

Statistical analysis was based on complete cases and guided by an analysis plan written according to the published trial protocol [21]. The analysis was conducted on a modified intention-to-treat (mITT) population of individuals who were PCR positive for *M. ulcerans* at enrolment, based on the randomised treatment allocation regardless of intervention received or whether it was wholly or partly taken.

Baseline demographic and other characteristics of participants were summarized using the mean and standard deviation for continuous variables, and counts with proportions for categorical variables. A generalized linear model assuming a normally distributed outcome was used to estimate mean differences in time to clearance of viable mycobacteria between intervention and control groups with associated 95% confidence intervals (CI). A modified Poisson regression model was used to estimate risk ratios with 95% confidence intervals for wound healing. Recurrence of BU, incidence of paradoxical reaction, and incidence of secondary infection were analyzed using a binomial regression or, for rare outcomes, exact logistic regression. Safety was assessed in all randomized participants receiving at least one treatment dose. Information on adverse events, serious adverse events (SAEs), and deaths was collected and reported in both arms of the trial.

## Results

The results of the study are reported according to the Consolidated Standards of Reporting Trials (S1 Checklist). Recruitment to the trial was stopped on 31^st^ July 2024, on advice of the DSMB and Trial Steering Committee (TSC) due to slow recruitment. Between 26^th^ November 2021 and 31^st^ July 2024, a total of 52 individuals were screened for inclusion into the study. In total, 42 individuals who met the inclusion criteria were randomized to receive either HR+DACC (23 participants) or SR+DACC (19 participants). Two participants in the SR+DACC arm were lost to follow-up at 20 weeks, and a further two participants in each arm were lost to follow-up by week 52. (Fig 1).

**Fig 1:**
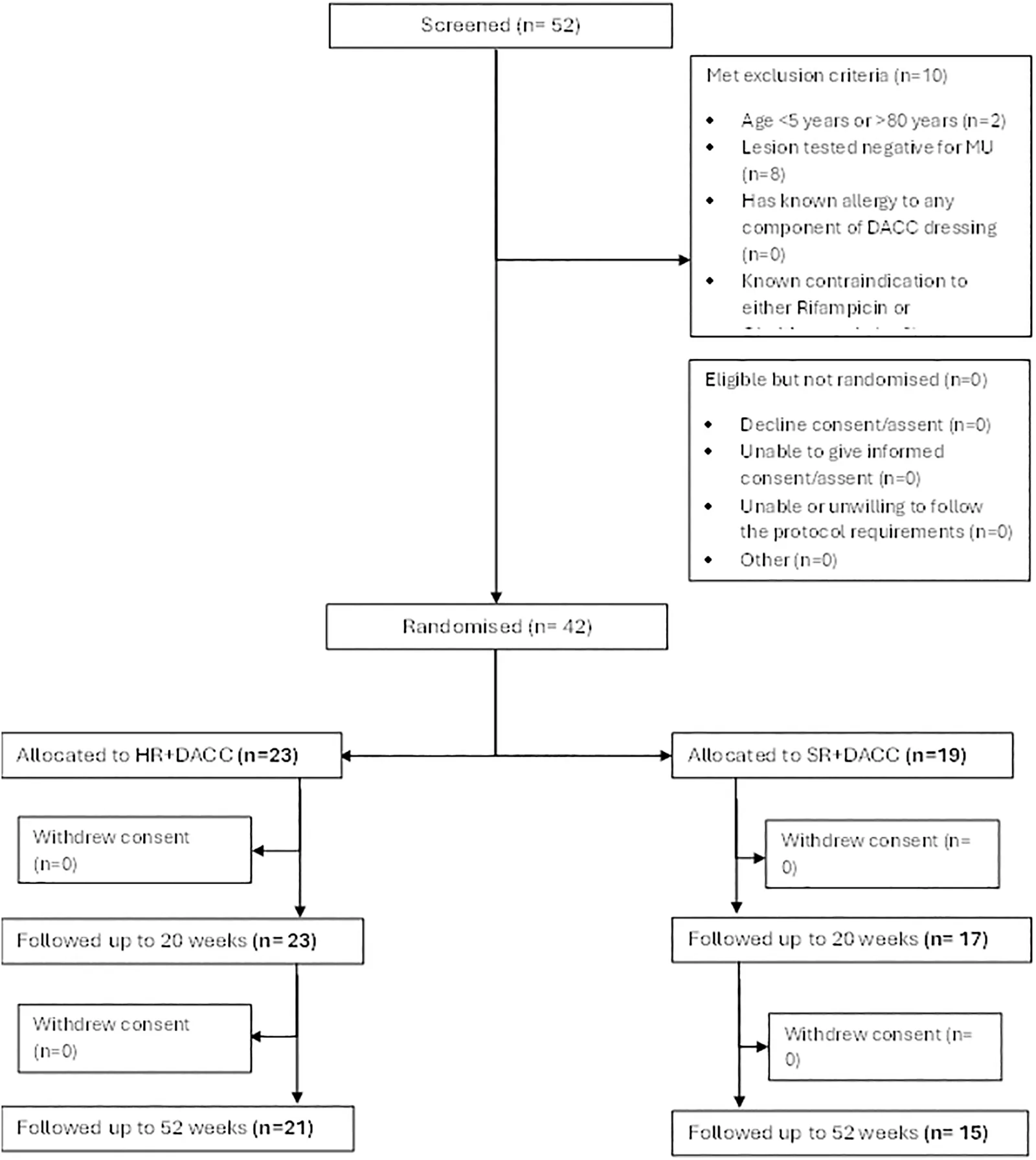
CONSORT Flow Diagram.

Participants in the HR arm were on average older, included more female participants, were slightly heavier, had lower levels of education, and were more likely to be in some form of employment. The distribution of participants was similar across arms in terms of the form of lesion (with ulcers being most common), stage of ulcer, and study sites (Table 1).

**Table 1:** Characteristics of participants at recruitment.

Characteristic	HR+DACC N = 23	SR+DACC N = 19	Total N = 42
Age in years, mean (SD)	35.9 (22.2)	19.6 (14.2)	28.5 (20.5)
Sex, n (%) Female Male	9 (39.1) 14 (60.9)	5 (26.3) 14 (73.7)	14 (33.3) 28 (66.7)
Weight in kilograms, mean (SD)	50.4 (14.8)	40.3 (16.4) 1 missing	46.0 (16.2)
Highest level of education, n (%)			
None	9 (39.2)	1 (5.3)	10 (23.8)
Primary	14 (60.9)	15 (79.0)	29 (69.1)
Secondary	0 (0.0)	3 (15.8)	3 (7.1)
Primary occupation, n (%)			
Unemployed	2 (8.7)	1 (5.3)	3 (7.1)
Student	6 (26.1)	10 (52.7)	16 (38.1)
Farmer	9 (39.1)	4 (21.1)	13 (31.0)
Trader	2 (8.7)	0 (0.0)	2 (4.8)
Miner	2 (8.7)	1 (5.3)	3 (7.1)
Other	2 (8.7)	3 (15.8)	5 (11.9)
Number of lesions, n (%)			
Single	16 (69.6)	12 (63.2)	28 (66.7)
Multiple	7 (30.4)	7 (36.8)	14 (33.3)
Form of lesions, n (%)			
Nodule	1 (4.4)	0 (0.0)	1 (2.4)
Plaque	0 (0.0)	1 (5.3)	1 (2.4)
Oedema	1 (4.4)	1 (5.3)	2 (4.8)
Ulcer	21 (91.3)	17 (89.5)	38 (90.5)
Stage of Buruli ulcer, n (%)			
Category I	4 (17.4)	3 (15.8)	7 (16.7)
Category II	3 (13.0)	4 (21.1)	7 (16.7)
Category III	16 (69.6)	12 (63.2)	28 (66.7)
Site, n (%)			
Pakro Health Centre	10 (43.5)	8 (42.1)	18 (42.9)
Wassa Amenfi East	13 (56.5)	11 (57.9)	24 (57.1)

HR+DACC: high-dose oral rifampicin with dialkylcarbamoyl chloride-coated dressings, the intervention group.

SR+DACC: standard-dose oral rifampicin with dialkylcarbamoyl chloride-coated dressings, the control group.

N: group size.

n: number of individuals in subgroup.

SD: standard deviation.

The mean time to clearance of viable *M. ulcerans* was 4.9 weeks in the HR arm versus 7.0 weeks in the SR arm, a crude difference of -2.1 weeks (95%CI -6.6 to 2.4), p-value 0.366. After adjusting for age, sex, stage of BU, and trial site, the mean difference was -0.5 weeks (95%CI -5.0 to 4.1, p-value 0.835).

Overall, there was a pattern suggesting more rapid microbiological clearance of *M. ulcerans* RNA in the HR arm. Participants in the HR achieved complete observed RNA negativity earlier, whereas those in the SR arm showed slower and less consistent clearance and remained RNA-positive in one tested sample at week 20 (Table 2).

**Table 2:** 16S rRNA positivity by study timepoint.

Study week	HR+DACC, n (%)	SR+DACC, n (%)
0	7/23 (30.43)	5/19 (26.32)
2	6/22 (27.27)	6/19 (31.58)
4	3/20 (15.00)	4/18 (22.22)
6	3/20 (15.00)	5/17 (29.41)
8	4/17 (23.53)	3/16 (18.75)
12	0/17 (0)	3/14 (21.43)
16	2/15 (13.33)	2/15 (13.33)
20	0/14 (0)	1/14 (7.14)

*Numerator represents the proportion of participants testing positive at the time point; denominator represents the total number of participants contributing a sample for testing at the time point. Positivity (%) = Number of RNA-positive samples/ Number of samples tested at that time point multiplied by 100.

Within 20 weeks of follow-up, wound healing occurred in seven of the 23 participants in the HR arm, and four of the 17 in the SR arm, a crude relative improvement of 29% (RR 1.29, 95%CI 0.44 to 3.77, p-value 0.637). After adjustment, the risk ratio was 0.88 (95%CI 0.25 to 3.13, p-value 0.849). No participants in either arm experienced recurrence of BU. Paradoxical reaction was observed in none of the participants in the HR arm [0% (0/21)] and five participants in the SR arm [33.3% (5/15)], a crude odds ratio of 0.08 (95%CI 0.00 to 0.65, p-value 0.016), which after adjustment was 0.19 (95%CI 0.00 to 0.35, p-value 0.102). Amongst participants followed up for up to 52 weeks, secondary infections occurred in 13 of the 21 participants in the HR arm, and 10 of the 15 participants in the SR arm, corresponding to a crude risk ratio of 0.93 (95%CI 0.56 to 1.56, p-value 0.770) and an adjusted risk ratio of 0.86 (95%CI 0.49 to 1.50) (Table 3).

**Table 3:** Effects of the intervention on primary and secondary outcomes.

Outcome	HR+DACC	SR+DACC	Unadjusted effect (95% confidence interval) p-value	Adjusted^1^ effect (95% confidence interval) p-value
*Assessed up to 20 weeks* ^2^	*N = 23*	*N = 17*		
Time in weeks to clearance of viable *M. ulcerans*, mean (SE)	4.9 (1.4) 1 missing	7.0 (1.9) 1 missing	MD -2.1 (-6.6 to 2.4) p = 0.366	MD -0.5 (-5.0 to 4.1) p = 0.835
Wound healing, n (%)	7 (30.4)	4 (23.5)	RR 1.29 (0.44 to 3.77) p = 0.637	RR 0.88 (0.25 to 3.13) p = 0.849
*Assessed up to 52 weeks* ^3^	*N = 21*	*N = 15*		
Recurrence of Buruli ulcer within 12 months, n (%)	0 (0.0)	0 (0.0)	_	_
Paradoxical reaction within 12 months, n (%)	0 (0.0)	5 (33.3)	OR 0.08 (0.00 to 0.65) p = 0.016	OR 0.19 (0.00 to 1.35) p = 0.102
Secondary infection within 12 months, n (%)	13 (61.9)	10 (66.7)	RR 0.93 (0.56 to 1.53) p = 0.770	RR 0.86 (0.49 to 1.50) p = 0.591

HR+DACC: high-dose oral rifampicin with dialkylcarbamoyl chloride-coated dressings, the intervention group.

SR+DACC: standard-dose oral rifampicin with dialkylcarbamoyl chloride-coated dressings, the control group.

N: group size.

n: number of individuals where different from overall group size.

SE: standard error.

p: p-value.

MD: mean difference.

OR: odds ratio.

RR: risk ratio.

^1^Adjusted for age, sex, stage of BU and trial site, except effect on paradoxical reaction which is only adjusted for age, sex and site due to low event counts.

^2^Excludes two participants in the SR+DACC arm who are lost to follow-up before week 20.

^3^Excludes two participants in the HR+DACC and a further two in the SR+DACC arm in addition to (2) above who are lost to follow-up before week 52.

No concomitant medications were prescribed or administered by the study team in either arm during the study. However, some participants reported the use of non-study medications, mainly occasional analgesics and ‘over-the-counter immune boosters’, which were documented in the Case Report Form (CRF) as concomitant medications and monitored throughout the study. All reported medications were reviewed for potential interactions with the investigational products, and no clinically relevant contraindications were identified; therefore, these medications were not considered to have affected study treatment or outcomes.

All reported adverse events were mild and resolved without additional treatment or intervention (Table 4). A total of 28 adverse events were reported among 23 participants across the two study arms during the study period: 15 events in 13 participants in the SR+DACC arm and 13 events in 10 participants in the HR+DACC arm. More than one adverse event was reported by four participants in the SR+DACC arm and two participants in the HR+DACC arm.

**Table 4:** Summary of adverse reactions.

Adverse Events	HR+DACC n (%)	SR+DACC n (%)	Total n (%)
**Participants with ≥1 adverse event**	**10**	**9**	**19**
**Total adverse events reported**	16 (52) *	15 (48) *	**31 (100)**
Serious adverse events	0	0	0
Non-serious adverse events	16 (100)	15 (100)	31 (100)
**Mucocutaneous/Dermatological manifestations**	**9 (56)**	**5 (33)**	**14 (45)**
Skin rash	1 (6) *	0	1 (3)
Body itch	1 (6) *	0	1 (3)
Itch on wound surface	7 (44) *	5 (33) *	12 (39)
**Constitutional symptoms**	**3 (19)**	**1 (7)**	**4 (13)**
Fever	0	1 (7) *	1 (3)
Dizziness	2 (13) *	0	2 (6)
Headache	1 (6) *	0	1 (3)
**Gastrointestinal symptoms**	**2 (13)**	**0**	**2 (6)**
Diarrhoea	1 (6)	0	1 (3)
Abdominal/Epigastric pain	1 (6) *	0	1 (3)
**Urine discolouration**	1 (6) *	7 (47) *	**8 (26)**
**Insomnia**	**1 (6)**	**0**	**1 (3)**
**Anorexia/Loss of appetite**	**0**	2 (13) *	**2 (6)**

HR+DACC: high-dose oral rifampicin with dialkylcarbamoyl chloride-coated dressings, the intervention group. SR+DACC: standard-dose oral rifampicin with dialkylcarbamoyl chloride-coated dressings, the control group. n: number of adverse events reported by participants in each arm. Percentages for specific adverse events are calculated using the total number of adverse events in each treatment arm as the denominator (HR+DACC: *n* = 16 adverse events; SR+DACC: *n* = 15 adverse events).

A total of 31 adverse events were reported in 19 participants. In the HR+DACC arm, four participants experienced multiple adverse events. One participant reported itchiness on the surface of the wound at week 4, followed by dizziness and epigastric pain at week 6; another participant experienced dizziness and headache concurrently at Week 4; a third participant reported body itch at Week 2 and two episodes of itch on the wound surface at weeks 6 and 12; and a fourth participant experienced urine discolouration and an itchy rash concurrently at week 2.

In the SR+DACC arm, two participants experienced multiple adverse events during follow-up. One participant reported recurrent urine discolouration at weeks 2, 4, and 12; and another participant experienced anorexia at week 2, fever and anorexia concurrently at Week 4, and itch on the surface of the wound at weeks 16 and 28.

## Discussion

In this randomized trial, we found a trend of faster time to clearance of viable *M. ulcerans* in the short-course high-dose arm (4.9 weeks vs. 7.0 weeks) with no evidence of a difference after adjustment. Wound healing rates at 20 weeks were similar between groups. Paradoxical reactions were observed only in the standard-dose arm (26.3% vs. 0%). Although paradoxical reactions occurred less frequently in the high-dose rifampicin arm, there was no evidence of a difference in their occurrence between the study arms and recurrence rates were zero in both groups. The trial was stopped early due to slow recruitment, which precludes definitive conclusions on the superiority of the high-dose rifampicin-based regimen, but it was well tolerated and potentially useful.

Our results are consistent with the possibility that a high-dose short course regimen could achieve comparable bacterial clearance to standard long-course therapy. While the adjusted difference was not statistically significant, the fact that the shorter regimen cleared the pathogen at least as rapidly as the longer regimen challenges the necessity of the full 8-week course [6]. Our findings add to the growing body of evidence suggesting that shorter-course antibiotic treatment regimens for Buruli ulcer may be feasible. Previous studies have reported the effectiveness of treatment durations shorter than the standard 8-week regimen in selected patients [17,28,29]. Our study provides additional evidence from a high-burden endemic setting that may support future evaluation of shortened treatment strategies.

The notably lower incidence of paradoxical reactions in the high-dose cohort is another important observation. Paradoxical reactions have been observed in 8–12% of Buruli ulcer patients in Africa during or after antibiotic treatment [30]. Patients experiencing PR tend to have a high bacterial load in baseline lesion samples and a greater likelihood of testing positive for *M. ulcerans* culture [31]. The marked reduction of paradoxical reactions in the HR arm may be attributed to shorter antigenic stimulation due to more rapid bacterial clearance in the high dose arm. Although the loss of statistical significance after adjustment, likely due to the small sample size and zero events in one arm, tempers any definitive conclusion, this finding constitutes an important indication in support of the potential benefits of the shorter regimen.

Consistent with evidence in tuberculosis [32,33], the safety profile of the high-dose regimen was reassuring. High-dose rifampicin was well tolerated, with no serious adverse events attributed to the higher dose. The types and frequency of adverse effects were similar between the two treatment arms, consistent with the extensive safety data from TB studies where similar or higher doses of rifampicin are used [33]. However, given the younger median age of our high-dose rifampicin cohort, these findings may not be generalizable to older endemic populations - notably those in Australia, where the median age of affected individuals approaches 60 years.

Our findings strongly support the need for a larger, adequately powered non-inferiority trial to definitively determine whether a 4-week high-dose regimen is as effective or superior to the current WHO recommended 8-week course. If confirmed in larger trials, a high-dose, short-course rifampicin regimen could have substantial clinical and public health benefits. Such a regimen would be particularly valuable in rural endemic regions where access to care is limited and prolonged treatment imposes a significant burden on affected individuals, families, and health systems, directly aligning with the WHO’s 2030 roadmap for NTDs.

## Supporting information

S1 Protocol BuruliRIFDACC Study Protocol. (PDF)

S1 Table Schedule of procedures before, during and after screening. (PDF)

S1 Checklist CONSORT 2025 checklist of information to include when reporting a randomised trial. (PDF)
