# Supplementary material for: High-dose rifampicin and clarithromycin for 4 weeks versus standard-dose rifampicin and clarithromycin for 8 weeks to treat Buruli ulcer: An open-label, individually randomized, controlled trial in Ghana
Source: PLoS Negl Trop Dis. 2026 Sep 29;20(9):e0014783. doi: 10.1371/journal.pntd.0014783 (PMC13645219; doi:10.1371/journal.pntd.0014783)
Supplement: Supplementary file 1 [file pntd.0014783.s001.pdf]

Study Protocol

A randomised controlled trial to evaluate the effect of High Dose Rifampicin and Dialkylcarbamoyl chloride (DACC)-coated dressings on outcomes in *Mycobacterium ulcerans* disease

**Version:**	**November 2020**
**Acronym:**	**BuruliRIFDACC**
**Study centre:**	**1) Ga West Municipal Hospital**
	**2) Pakro Health Centre**
	**3) Wassa Akropong Municipal Hospital**
**Study design:**	**Phase 3 Prospective randomised open-blinded end-point (PROBE) study**
**Study area:**	**Ghana – West Africa**
**Sponsor:**	**London School of Hygiene & Tropical Medicine**
**Funding:**	**NIHR Research and Innovation for Global Health Transformation**
**Grant Number:**	**NIHR200125**
**Protocol Number:**	
**PACTR Number:**	**PACTR202011867644311**
**LSHTM Ethics Reference:**	**22912**
**GHS Ethics Reference:**	**GHS-ERC 003/01/21**
**Proposed start date:**	**March 2021**
**Proposed end date:**	**September 2023**

The Information contained herein is the property of the Kwame Nkrumah University of Science & Technology, Kumasi (KNUST), the Kumasi Centre for Collaborative Research (KCCR), the Noguchi Memorial Institute for Medical Research (NMIMR) and the London School of Hygiene & Tropical Medicine (LSHTM). Its contents may not be reproduced, published or disclosed to others without the written authorisation of the above -mentioned institutes.

TABLE OF CONTENTS

List of abbreviations *4*

TEAM ROSTER *6*

1.0 INTRODUCTION *14*

1.1 Background **14**

1.2 Rationale **14**

2.0 OBJECTIVES AND ENDPOINTS *18*

2.3 Secondary objectives **18**

3.1 Overall design **19**

3.3 Group allocation **20**

3.4 Economic evaluation **20**

3.5 Milestones **21**

4.1 Inclusion criteria **22**

4.2 Medications **22**

4.3 OTHER MEDICATIONS **22**

4.3.1 Medication(s)/treatment(s) permitted before and/or during the trial **22**

4.3.3 CONTRAINDICATIONS TO RIFAMPICIN **23**

4.4 Participant Withdrawal criteria **25**

5.1 Sensitisation **26**

5.2 Screening and recruitment **26**

5.2.2 Initial Sample collection and confirmation **28**

5.3 Data Collection: **29**

5.3.1 Clinical Data Collection **29**

5.3.2 Economic and quality of life data collection **30**

5.4 Adverse Events: **31**

5.4.1 Assessment procedures **32**

5.5 Reporting **32**

5.5.1 Serious Adverse Reactions (SARs)/Serious Adverse Events (SAEs) **33**

5.5.2 SUSARs **33**

5.6 Urgent safety reporting **33**

5.7 Quality control and validation **34**

6 ANALYSIS & STATISTICAL CONSIDERATIONS *35*

6.1 General design **35**

6.2 Sample size justification **35**
6.3 Statistical methods **35**
6.4 Populations to be analysed **36**
6.5 Efficacy Endpoints **37**
Primary Endpoint: **37**
6.6 Economic Evaluation **38**
7.0 DATA MANAGEMENT AND MONITORING ***41***
7.1 Source documentation **41**
7.2 Study documentation and data safety **41**
7.3 Data entry and storage **40**
7.4 Study monitoring **40**
8.0 Results **42**
9.1 Research Ethics Committee **43**
9.2 Informed Consent Form (ICF) **43**
9.3 Benefits **44**
9.4 Risks **44**
9.5 Sample and data ownerships: **44**
9.6 Compensation **44**
9.7 Study registration **45**
9.8 Declaration of interests **45**
9.9 Audits and Inspections **45**
9.10 Indemnity **45**
9.11 Quality Control and Validation **45**

List of abbreviations

AMR	Antimicrobial Resistant
BU	Buruli Ulcer
BUD	Buruli Ulcer Disease
BUFLS	Buruli Ulcer Functional Limitation Score
CRF	Case Report Form
DACC	Dialkylcarbamoyl chloride
DRB	Dry Reagent Based
FDA	Federal Drug Authority
FNA	Fine Needle Aspirate
GCP	Good Clinical Practice
ICF	Informed Consent Form
IDM	Intensified Disease Management
ITT	intention to treat
KCCR	Kumasi Centre for Collaborative Research in Tropical Medicine
KNUST	Kwame Nkrumah University of Science & Technology
LSHTM	London School of Hygiene & Tropical Medicine
MU	*Mycobacterium ulcerans*
NIHR	National Institute of Health Research (United Kingdom)
NMIMR	Noguchi Memorial Institute of Medical Research
NTD	Neglected Tropical Diseases
PAR	Percentage area reduction
PCR	Polymerase Chain Reaction
RC	Rifampicin / Clarithromycin
RIGHT	Research and Innovation for Global Health Transformation
RT qPCR	Real Time Quantitative Polymerase Chain Reaction
SAP	Systems application and products
SOC	Standards of care
SOP	Standard operating procedure
VG	Vaseline gauze
WHO	World Health Organisation

Protocol Approval Signatures

The signatures below document the approval of this protocol and the attachments, and provide the necessary assurances that this study will be conducted according to all stipulations of the protocol, including all statements regarding confidentiality and according to local legal and regulatory requirements. They will also ensure that only the approved version of the protocol is available at the study sites as per Good Clinical Practice (GCP) regulations.

Sponsor/Sponsor Delegated Person (SDP):

Patricia Henley	Signature	Date
		1/12/2020

Principal Investigator:

Prof Dr Richard Odame Phillips	Signature	Date
		1/12/2020

Co -investigator

Dr Michael Marks	Signature	Date
		1/12/2020

**Senior Statistician**	Signature	Date
Prof Elizabeth Allen		1/12/2020

Senior Coordinating Physician

Dr. Yaw Ampem Amoako	Signature	Date
		1/12/2020

Trial Clinician:

Dr. Joseph Tuffour	Signature	Date
		1/12/2020

Trial Coordinator:

Ms. Abigail Agbanyo	Signature	Date
		1/12/2020

TEAM ROSTER

**Investigators/contact details**	
**Ghana**	**Prof Richard Phillips, MBChB, PhD, FWACP, FGCP**<br><br>Kumasi Centre for Collaborative Research (KCCR) at the Kwame Nkrumah University of Science and Technology (KNUST), Kumasi, Ghana<br><br>Tel: +233-209140451<br><br>e-mail: phillips@kccr.de
**United Kingdom**	**Dr Michael Marks**<br>Clinical Research Department<br>London School of Hygiene & Tropical Medicine (LSHTM)<br>Keppel Street<br><br>London WC1E 6JT<br>Email: michael.marks@LSHTM.ac.uk

Study Coordinator: Ms. Abigail Agbanyo

**Pharmacist**	Dr Happy Ama Amedzro
**Local Trial Monitoring**<br><br><br>**Independent Trial Monitor**	Bernadette Agbavor<br><br><br>Carl Kwame Adoku
**Sponsor's Central Laboratories**<br><br>**Trial Laboratory lead**	Kumasi Centre for Collaborative Research (KCCR)<br>Noguchi Memorial Institute of Medical Research (NMIMR)<br><br>Dr Michael Frimpong
**Trial Statistician**	Prof Elizabeth Allen
**Field Coordinators**	Isaac Doku<br>Clement Tettey
**Trial Clinicians**	Dr. Yaw Ampem Amoako

	Dr. Joseph Tuffour

**Trial Steering Committee**	Dr Kingsley Asiedu<br><br>Dr. Michael Marks<br><br>Dr Edwin Ampadu<br><br>Dr Albert Paintsil<br><br>Mr Emmanuel Agumah
**Data Safety Monitoring Board**	Dr Abraham Oduro<br><br>Prof Yaw Asante Awuku<br><br>Dr Ansumana Bockarie<br><br>**Dr. Christian Holm Hansen**

**Funding Support**	
	**UK National Institute for Health Research (NIHR)**<br><br>**Research and Innovation for Global Health Transformation (RIGHT) programme**<br><br>Web: www.nihr.ac.uk

*This study has been approved for funding by the NIHR.

Clinical Trial Sponsor
LSHTM, Keppel Street, London, United Kingdom

Sponsor Delegated Person: Patricia Henley

Trial Management
The Co-Principal Investigators at KCCR and LSHTM and the Trial Management Group (TMG) will provide oversight for the day-to-day management of the trial. A trial coordination office will be located at the Kumasi Centre for collaborative research (KCCR) at the KNUST in Kumasi. Ms Abigail Agbanyo will be responsible for the day-to day coordination of the trial.

PROTOCOL SYNOPSIS

**Protocol Title**	**A randomised controlled trial to evaluate the effect of High Dose Rifampicin and Dialkylcarbamoyl chloride (DACC)-coated dressings on outcomes in *Mycobacterium ulcerans* disease**
**Short Title**	High-RIF and DACC for treating *Mycobacterium ulcerans* disease
**Trial acronym**	BuruliRIFDACC
**Health Condition**	Mycobacterium ulcerans diseases (Buruli ulcer)
**Study Centre**	**1) Ga West Muncipal Hospital**<br>**2) Pakro Health Centre**<br>**3) Wassa Akropong Municipal Hospital**
**Interventions**	Intervention:<br>High Dose (20mg/kg) **oral** Rifampicin, standard dose **oral** clarithromycin (15mg/kg) for four weeks and **topical** Dialkylcarbamoyl chloride (DACC)-coated dressings for four weeks<br><br>Control:<br>Standard dose **oral** Rifampicin (10mg/kg), standard dose **oral** clarithromycin (15mg/kg) and **topical** Dialkylcarbamoyl chloride (DACC)-coated dressings for eight weeks<br><br>All participants will receive an application of short stretch bandaging to reduce surrounding oedema and functional limitation assessment.<br><br>Treatment will be administered during patient scheduled visits and this will be monitored by trained community-based volunteers and health workers in the various community-based health centers.
**Trial Design**	Individually randomised controlled trial
**Trial objectives**	Primary Objective<br>• Compare the time to clearance of viable Mycobacterium from wounds of patients treated with high-dose **oral** rifampicin and **topical** DACC dressings (HR-DACC) to those receiving standard dose **oral** rifampicin and **topical** DACC dressings

	(SR-DACC).<br>Secondary Objectives<br>• Assess impact of high-dose Rifampicin and DACC dressings on the proportion of wounds healed at 20 weeks<br>• Assess the difference in proportions of paradoxical reactions between groups<br>• Asses the difference in proportions of recurrence between groups<br>• Use historic control data to compare the time to clearance of viable Mycobacterium in patients receiving standard dose Rifampicin and Vaseline gauze dressings.<br>• Evaluate the incremental costs and cost-effectiveness of high dose Rifampicin vs standard dose Rifampicin
**Trial Endpoints**	Primary efficacy endpoint:<br>Time to clearance of viable mycobacterium from wounds. **This would be evaluated at week 8.**<br><br>Secondary endpoints:<br>• Time to healing of the BU wound between groups<br>• Proportion of patients with paradoxical reaction in each treatment arm<br>• Proportion of patients with recurrence of BU in each treatment arm<br>• Proportion of patients with clinical evidence of a secondary infection during their BU treatment<br>• The incremental financial and economic costs per patient of the high dose Rifampicin vs standard dose Rifampicin (from household and provider perspectives)
**Participant number**	112 participants
**Inclusion criteria**	i. Age between 5 and 80 years old<br>ii. Have a nodule, plaque or ulcer, with or without associated oedema.<br>iii. Lesion that has tested positive for *M. ulcerans* by Polymerase Chain Reaction (PCR) for IS2404

	iv. Participant or parent/guardian able to give informed consent.<br>v. **Participant or parent/guardian able to give assent (13-17 years)**<br>vi. Able and willing to follow the protocol requirements
**Exclusion criteria**	i. Current participation in any interventional study at the time of randomisation<br>ii. Known allergy to any component of DACC dressing<br>iii. Known contraindication to either Rifampicin or clarithromycin<br>iv. **Pregnant women**
**Statistical Rationale**	Primary analysis will be carried out on groups as randomised ('intention to treat', ITT).<br><br>All analyses will account for the nature of the distribution of the outcome and results will be presented as appropriate effects sizes (mean difference between arms and risk ratios) with a measure of precision (95% confidence intervals)<br><br>**Safety Analysis**<br>An independent Data Safety and Monitoring Board (DSMB) will be established to review, in strict confidence, data from the trial. Information of all adverse events and deaths will be collected and reported in both arms of the trial.<br><br>One scheduled interim analysis will be performed by an independent statistician once approximately half of the target population has achieved the minimum duration of study follow-up.
**Trial procedures**	Patients will be followed to at least Month 5.<br>Visit 1: Screening & Baseline<br>Visit 2: Treatment (week 2)<br>Visit 3: Treatment (week 4)<br>Visit 4: Treatment (week 6)<br>Visit 5: Treatment (week 8)<br>Visit 6: Follow up at month 3<br>Visit 7: Follow up at month 4<br>Visit 8: Follow up at month 5<br>Visit 9: Follow up at month 6<br>Visit 10: Follow up at month 7<br>Visit 11: Follow up at month 8<br>Visit 12: Follow up at month 9<br>Visit 13: Follow up at month 10<br>Visit 14: Follow up at month 11

	Visit 15: Follow up at month 12
**Study Timeline**	Estimated date of first participant enrolment: Quarter Q1/2021<br>Estimated date of last participant enrolment: Quarter Q4/2022<br>Estimated date of last participant completed: Quarter Q2/2023<br>**Estimated date for analysis completed: Quarter Q4/2023**<br><br>Total duration of Study= **30 months**<br><br>**(23 months enrolment + minimum 5 months follow-up after last patient enrolment + 6 months analysis period)**<br><br>**Anticipated study duration is from beginning of March 2021 to end of September 2023**

Schedules of Procedures before (screening), during and after treatment

Activity	**Screening**	**Week 0**	**Week 2**	**Week 4**	**Week 6**	**Week 8**	**Monthly**	**12 Months**	**Treatmet Failure**
Written Informed Consent	X								
Inclusion / Exclusion Criteria	X	X							
Randomisation		X							
Socio-demographics	X	X							
History and Physical Exams		X	X	X	X	X	X	X	X
Clinical Assessment	X	X	X	X	X	X	X	X	X
Adverse Events			X	X	X	X	X	X	X
Clinical Assessment of the Patient and Lesion		X	X	X	X	X	X	X	X
Measurement of Lesion Size		X	X	X	X	X	X	X	X
Photograph of lesion by digital camera	X	X	X	X	X	X	X	X	X
Counselling and testing for Pregnancy*	X								
Swab/FNA for PCR	X	X							X
Swab/FNA for 16S rRNA (0, 2, 4, 6, 8 weeks and then monthly if not healed)		X	X	X	X	X	X		X
VAS		X	X	X	X	X	X	X	X
Functional limitations assessment		X	X	X	X	X	X	X	X
Health-related quality of life (QALY, DALY) for economic evaluation		X	X	X	X	X	X	X	X
Household costs of BU and of care seeking		X	X	X	X	X	X	X	
Socioeconomic status		X	X	X					

Individuals will be followed for a minimum of twenty weeks. Visits beyond this will be conducted within the framework of routine care.

* Only on female patients aged 10-50 years using **Urine Pregnancy Test (UPT) and information on the last menstrual period Counselling will also be given to all females of reproductive age who are not pregnant at week 0 on the need for contraception (including barrier methods such as female and/or male condoms) and avoidance of pregnancy during the period of taking the study medication.**

1.0 INTRODUCTION

1.1 Background

Buruli ulcer (BU) is a neglected tropical disease (NTD) caused by infection with *Mycobacterium ulcerans* (MU) and occurs in more than 30 countries worldwide with a focus in rural parts of West African countries including Ghana[1]. A national survey conducted in 1999 reported over 6000 cases and since 2005, 35 districts from 6 regions (Ashanti, Brong Ahafo, Central, Eastern, Greater Accra and Western) in Ghana have reported an average of about 1000 cases per year[1]. It manifests as large, disfiguring skin ulcers mainly in children aged 5 to 15 years, although it can affect any age[2]. Access to treatment in rural areas can be challenging and late presentation is typical, due to fear, stigma, suspicion about conventional medicine and economic consequences for poor families[3,4] Geographically, the disease is highly focal and in Ghana, for example, it is confined to the south, and particularly to parts of the Ashanti Region. Whilst the establishment of the World Health Organization (WHO) Buruli ulcer initiative in 1998, together with improved diagnosis and management, brought major advances in understanding of the disease mechanism, the mode of transmission remains unknown.

Initial lesions manifest as either a subcutaneous painless nodule tethered to the skin or an intradermal plaque. These enlarge over a period of days to weeks, ulcerating in the centre. Ulcers are painless, with a necrotic base and irregular, undermined edges. Surrounding oedema occurs in about 10% of cases. Ulcers enlarge progressively and may cover the whole of a limb or the trunk if left untreated, although the patient remains systemically well unless a secondary bacterial infection occurs[5].

Central to the pathogenesis of MU is production of a lipid cytotoxin called mycolactone, which at low concentration is immunosuppressive and inhibits chemotaxis[6,7]. Histology of Buruli lesions shows clusters of extracellular MU surrounded by necrotic subcutaneous fat, suggesting that mycolactone diffusing from the organisms is responsible for tissue damage.

Antibiotic treatment introduced over the last fifteen years has transformed the outlook for patients with BU. However, the currently recommended regimen of oral rifampicin (10mg/kg dose) together with intramuscular streptomycin or clarithromycin for 8 weeks is far from ideal, particularly given the increasing global threat of antimicrobial resistance (AMR)[8]. Although the disease can be cured in most patients who adhere to this regimen, healing rates are highly variable even in patients with seemingly similar lesions. Previous studies have reported median healing times for nodules of 8 weeks and for category I lesions of 12-18 weeks, and 15-30 weeks for category II and III lesions[8–10] and that this is similar regardless of the antibiotics regime used. Available data from various studies also suggest that healing of up to two thirds of patients occurs within 25 weeks after onset of treatment.

1.2 Rationale

WHO classifies BU as one of five NTDs in line for the “innovative and intensified disease management (IDM)” approach, requiring a major scaling up of active case detection, treatment, monitoring and surveillance. Clinical trials led in Ghana have led to 8 weeks of Rifampicin and Clarithromycin being established as the standard of care[8].

As outlined above, wound healing remains prolonged in BU despite the use of effective antimicrobial therapy. Preliminary evidence from studies in Ghana indicates two main factors contributing to variability in healing rate, i) the bacterial load at baseline and ii) the

development of paradoxical reactions, with renewed inflammation, despite progressive bacterial killing by antibiotics. Previous studies have demonstrated either healing or lack of detectable live MU in 46% of patients by week 4 of treatment, suggesting that a shorter course of antibiotics may suffice for some patients, whereas, in those with unhealed lesions at week 8, more than 50% had detectable live organisms using the combined 16S rRNA assay and qPCR for IS2404[9].

An additional factor that needs consideration is the possible role of secondary infections of BU wounds. It has been proposed that mycolactone may limit occurrence of secondary infections in BU lesions, but supporting evidence is largely missing. In fact, a number of studies in Ghana have reported that diverse microbial communities can be isolated within BU lesions and there is some evidence that clinicians prescribe additional antibiotics to treat these secondary infections. However, whether these other bacteria isolated from BU lesions represent true co-infections, secondary colonisation or opportunistic commensals remains unclear[5,11]. As highlighted, presumed secondary infections may contribute to antimicrobial use and potentially to AMR, highlighting the need to understand if and how they interfere with BU resolution.

Several approaches may be taken to improving outcomes in patients with BU. One approach is to enhance antimicrobial therapy to improve the clearance of viable mycobacterium from wounds. Rifampicin is the mainstay drug of the regimes used to treat BU. Current treatment guidelines use a dose of 10mg/kg, which was selected in line with standard doses used in the treatment of tuberculosis. There is now considerable interest in using higher doses of Rifampicin in the treatment of mycobacterial infections including both Tuberculosis and Buruli Ulcer. Multiple studies have demonstrated that higher dose Rifampicin is safe and well tolerated[12,13], and multiple large-scale trials of this strategy are underway in tuberculosis. Preliminary data for Buruli Ulcer also suggests that higher dose Rifampicin may improve outcomes and may facilitate shortening of regimes in part because they are may clear viable mycobacterium more quickly[14]. Collectively, these data suggest that high-dose rifampicin is likely to be a safe and efficacious strategy to improve outcomes for patients with BU; however, its use has not been evaluated in a robust clinical trial.

A second approach to tackling both the consequences of high mycobacterial load in wounds and secondary infections would be a strategy to directly target and remove both MU and other bacteria from the wound. Dialkylcarbamoyl chloride (DACC)-coated dressings irreversibly bind bacteria on wound surfaces resulting in their removal when dressings are changed. They represent an innovative approach to prevention of infection without use of antimicrobials. A systematic review of evaluations of DACC dressings on wound healing was published in 2017[15]. A total of seventeen studies, including 3,408 patients were included in the systematic review. All studies amongst patients with chronic wounds used an active comparator of silver impregnated dressings. Unlike plain gauze dressings used for BU care in Ghana, silver-impregnated dressings have a known antibacterial-effect. A single RCT and two cohort studies all demonstrated that DACC dressings reduced bacterial load to a greater extent than silver-impregnated dressings and one RCT also demonstrated a positive effect on wound size reduction. Based on these data we would anticipate that DACC dressings would also perform better than the plain gauze dressings, which are routinely used in Ghana.

In addition to their impact on secondary infections, our group has demonstrated that MU itself binds directly to DACC dressings and is removed during dressing changes[16]. As such, regular dressing changes represent a pathway to directly reducing the bacterial burden with BU lesions which would be anticipated to improve healing of BU. DACC dressings have also been tested

in uncontrolled studies in both Ghana (Frimpong M, personal communication) and Australia (Larsson I, personal communication) with promising results. Therefore, overall DACC dressings potentially offer multimodal mechanisms to improve BU wound healing by directly reducing mycobacterial load, potentially reducing paradoxical reactions and reducing secondary infections. An additional benefit would be a reduction in use of antimicrobials for treatment of secondary infections. By improving wound healing, we anticipate improved outcomes and reduced disability for the patient and reduced health care costs for the health system which may, in turn, contribute to reductions in stigma and earlier care-seeking, allowing more patients to benefit from effective treatment.

Overall, these data suggest that combining high-dose Rifampicin (HR) and DACC dressings may significantly improve the time to clearance of viable mycobacterium from wounds, reduce the risk of paradoxical reactions and secondary infections and therefore improve outcomes for patients with BU.

This study will determine the efficacy of combining high-dose **oral** Rifampicin and **topical** DACC dressing by comparing:

- High-dose Rifampicin (HR) combined with standard-dose clarithromycin and use of a DACC dressings applied on alternate days to lesions (HR+DACC)
  to
- Standard-dose Rifampicin (**SR**) and clarithromycin and use of a DACC dressing applied on alternate days to lesions (**SR**+DACC)

In addition, an economic evaluation will be conducted to assess the **incremental costs and cost-effectiveness** of switching from standard BU treatment strategies to either HR+DACC or SR+DACC by combining data from this trial with existing data from previous BU trials. While the higher dose of Rifampicin and DACC dressings themselves are expected to cost more than the standard treatment, the less frequent dressing changes (alternate days with topical DACC vs. daily with standard care), faster healing times, and reduced rates of secondary infections are expected to result in fewer contacts between patients and clinicians/Community based surveillance volunteers (CBSVs) and fewer dressings and other resources used. It is therefore, unclear whether the overall costs of the high-dose Rifampicin and DACC dressing strategy will be greater or less than standard care.

Success in this project would have a major impact on the management of Buruli ulcer, with faster healing leading to shorter treatment duration for many patients. Further, economic and social benefits could be significant. Reducing the use of antibiotics to treat presumed secondary infections could result in significant cost savings to the healthcare system and additionally provide the opportunity for patients to receive more of their treatment **at the hospitals and various health centers at the community level.**

2.0 OBJECTIVES AND ENDPOINTS

The aim of the study is to determine the ability of high-dose **oral** Rifampicin (20mg/kg) and DACC dressings to improve the time to clearance of viable mycobacterium from Buruli ulcer wounds compared to a regime using standard dose **oral** Rifampicin (10mg/kg) and DACC dressings.

2.1 **Study Hypothesis**

The main hypothesis of the study is that the combination of high dose Rifampicin and DACC dressings will improve clearance of mycobacterium and wound healing. We hypothesise that this effect will be mediated by i) faster killing of MU amongst participants; ii) reduction in the number of patients with paradoxical reactions; and iii) reduction in the number of secondary infections. We hypothesise that this combined HR+DACC strategy may be cost-effective and potentially even cost-saving relative to standard care.

2.2 Primary objective

- Compare the time to clearance of viable Mycobacterium from wounds of patients treated with high-dose **oral** Rifampicin and DACC dressings (HR+DACC) to those receiving standard dose **oral** Rifampicin and DACC dressings (SR+DACC). **This will be evaluated at week 8. Samples will be collected to evaluated for viable mycobacterium at week 2,4,8 and if unhealed 12 and 16.**

2.3 Secondary objectives

- Assess impact of high-dose Rifampicin and DACC dressings on the proportion of wounds healed at 20 weeks
- Assess the difference in proportions of paradoxical reactions between groups
- Asses the difference in proportions of recurrence rate in both groups
- Use historic control data to compare the time to clearance of viable Mycobacterium in patients receiving standard dose Rifampicin and Vaseline gauze dressings.
- Evaluate the incremental costs and cost-effectiveness of High dose Rifampicin+ DACC dressings vs Standard dose Rifampicin + DACC to historic controls receiving standard dose Rifampicin and Vaseline gauze dressings

3.0 STUDY SETTING AND DESIGN

3.1 Overall design

This is an individual randomised, multi-centre, randomised trial of high-dose Rifampicin and DACC dressing, compared to standard-dose Rifampicin and DACC dressings. Participants will be recruited at a range of BU treatment centres: Ga West Municipal Hospital, Pakro Health Centre and Wassa Akropong Municipal Hospital. All these hospitals serve surrounding endemic communities whose main occupation is farming and mining.

The design will compare care using high-dose Rifampicin (20mg/kg), clarithromycin (15mg/kg) and DACC dressings to standard-dose Rifampicin (10mg/kg), clarithromycin (15mg/kg) and DACC dressings.

Data for the trial efficacy endpoints are collected at 8 and 20 weeks post randomisation. Both groups will receive longer term follow-up in line with national guidelines. Additional data collected after 20 weeks, up to the time at which the last participant randomised has reached 20 weeks follow up, will be used in the health economic evaluation. We expect approximately 50% of randomised participants to reach the 12 month follow up visit during the conduct of the trial. The period of time covering trial recruitment up to the last participant's 20 week visit will be referred to as the study duration and is expected to be approximately 19 months.

groups will receive follow-up in line with national guidelines.

3.2 Study sites Ghana

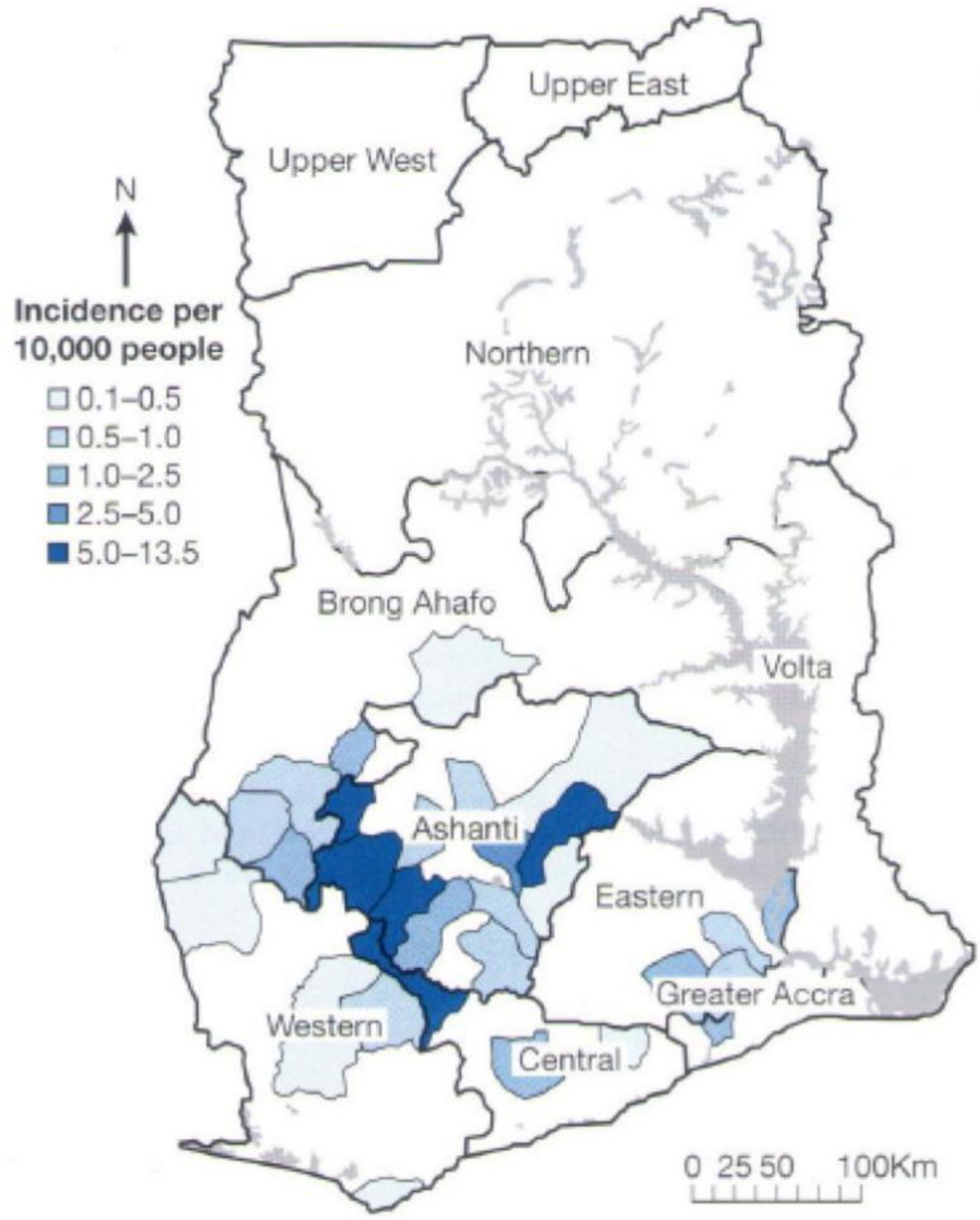

The study area of Ghana were selected based on most recent figures of BU prevalences, provided by the National Buruli Ulcer Control Programme, and because they are not participating in other studies on Buruli Ulcer at this time to avoid reducing the potential recruitment population. .

Fig.1 Buruli ulcer endemic districts in Ghana

3.3 Group allocation

Randomisation

Participants enrolled in the study will be allocated 1:1 to one of the two treatment groups following computer-generated permuted block randomisation with stratification by site and stage of BU (Stage I to III). Each participant will receive a unique study code, kept with the local site team and the study coordinators.

Interventions

The two groups will receive either of the following interventions:

- High-dose **oral** Rifampicin (20mg/kg), standard dose **oral** clarithromycin (15mg/kg) for 4 weeks and DACC dressing applied to the wound and changed every 48 hours **(HR+DACC)** until the lesion heals.

Or

- Standard-dose **oral** Rifampicin (10mg/kg), standard dose **oral** clarithromycin (15mg/kg) for 8 weeks and DACC dressing applied to the wound and changed every 48 hours until lesion heals.
  (SR+DACC)

All participants will receive an application of short stretch bandaging to reduce surrounding oedema as well as assessment for functional limitation by the Buruli Ulcer Functional Limitation Score (BUFLS) as recommended by WHO.

3.4 Economic evaluation

The incremental costs, cost-effectiveness, and equity of using high-dose Rifampicin and DACC wound dressings relative to alternative strategies will be assessed from provider, household, and societal (i.e. combined provider and household) perspectives, consistent with reference case specifications[17]. Both financial costs, which reflect actual monies paid, and economic costs, which reflect the full value of resources used – including, for example, patient, volunteer, or clinician time and donated resources – will be assessed. Both the costs of treatment (from household and provider perspectives) and of living with BU (from household perspective) will be assessed, including productivity losses in cases where having BU prevents the patient and/or their carer from participating in usual productive activities. All costs pertaining to research activities (i.e. data collection which does not affect patient outcomes) will be excluded. We will include both the recurrent costs associated with each patient treated, and also the "start-up costs" incurred by providers in switching from current practice to high-dose Rifampicin and DACC dressings; these start-up costs are likely to include meetings, training, and preparation of any training materials.

We will assess actual practice in the trial and develop a model to explore how costs and effects may be expected to vary outside the trial setting in a "real-world" context. If there is evidence for differences in costs and/or health outcomes beyond duration of the study, the model will also reflect a lifetime horizon. The model will draw on data collected as part of the trial and longer term follow up and combine it with additional data sources to inform decisions regarding a potential switch in policy to a treatment strategy incorporating high-dose Rifampicin and DACC dressings.

3.5 Milestones

- Preparation of all trial related SOPs, operational manuals, clinical operations plans, communications plan, training manuals, study risk log, and setting up of databases.
- Obtaining Ethical Approval from the following Ethics committees:
    i. *Committee on Human Research Publication and Ethics of the School of Medical Sciences, Kwame Nkrumah University of Science and Technology, Kumasi, Ghana.*
    ii. *Interventional Research Committee, London School of Hygiene & Tropical Medicine, London, United Kingdom*
- Obtaining clinical trial study approval from appropriate authorities.
- Agreement with the local health authorities, district medical officers and community leaders in the selected study Districts
- Training of scientists and village volunteers
- Obtaining informed consent / assents (from parents of patients under 18 years) from study participants
- Enrolment of eligible participants and documentation
- Laboratory assessments and data management
- Follow up of patients and site monitoring
- Finalisation of laboratory assessments and data files
- Final assessment of results and statistical analysis
- Information regarding results to local health authorities and participants
- Publication of results
- Final report to the funding agency and the regulatory bodies including FDA Ghana (before publication of results as per the guidelines for authorisation and the conduct of clinical trials).

4.0 SELECTION AND WITHDRAWAL OF PARTICIPANTS

Patients will be provided with a full explanation of the nature, purpose and requirements of the study including information sheets and informed consent forms. They will be invited to participate in a screening evaluation, which will include a medical history, physical examination and wound assessment. The specific inclusion and exclusion criteria are indicated below.

4.1 Inclusion criteria

i. Ages between 5 years and 80 years
ii. Has a nodule, plaque or ulcer, with or without associated oedema
iii. Lesion must have tested positive for Mu by PCR for IS2404
iv. Able to give informed consent or parent/guardian able to give informed consent
v. **Participant or parent/guardian able to give assent (13-17 years)**
vi. Able and willing to follow the protocol requirements

Exclusion criteria

i. Current participation in any interventional study at the time of randomisation
ii. Known allergy to any component of DACC dressing dressing
iii. Known contraindication to either Rifampicin or Clarithromycin
iv. Pregnant women

4.2 Medications

All patients who test positive for MU by PCR for IS2404 will receive either high-dose **oral** Rifampicin (20mg/kg) or standard dose of Rifampicin (10 mg/kg). **All participants will receive standard dose oral clarithromycin (15mg/kg) daily for 4 weeks for the intervention group and 8 weeks for the control group. The source of study drugs; Rifampicin and Clarithromycin will be supplied by the WHO routine care as part of the National BU Control programme.**
In addition, study participants will all receive **topical** DACC dressings. DACC dressing comprises an easy-to-apply two-layer system. **The medications have been summarized in the treatment schedule below. Details will also be provided in the accompanying SOP.**
The study investigators will be able to tell the number of pills you take based on your body weight. The schedule of treatment has been put in the table below.

Treatment schedule

<table>
<tr><th colspan="9">Oral Medication</th><th rowspan="2">Topical DACC</th></tr>
<tr><th rowspan="2">Arm of study</th><th colspan="8">Patient's Visit</th></tr>
<tr><th>Week 1</th><th>Week 2</th><th>Week 3</th><th>Week 4</th><th>Week 5</th><th>Week 6</th><th>Week 7</th><th>Week 8</th><th rowspan="3">Every 48 hours until lesion heals</th></tr>
<tr><td>HR (20mg/kg Rifampicin + 15mg/kg Clarithromycin) + DACC</td><td>X</td><td>X</td><td>X</td><td>X</td><td></td><td></td><td></td><td></td></tr>
<tr><td>SR (10mg/kg Rifampicin + 15mg/kg</td><td>X</td><td>X</td><td>X</td><td>X</td><td>X</td><td>X</td><td>X</td><td>X</td></tr>
</table>

**Clarithromycin) + DACC**									

These drugs come in pills and the study investigators will be able to tell the number of pills the participant has to take depending on body weight as elaborated in the table below.

Dosage schedule

	**Dosage Strength**					
	**Clarithromycin (tablets)**		**Standard Rif (SR)(capsules)**		**High Rif (HR)(capsules)**	
**Weight (kg)**	**500mg**	**250mg**	**300mg**	**150mg**	**300mg**	**150mg**
**20-39**	**1**	**2**	**1**	**2**	**2**	**4***
**40-54**	**1**	**2**	**1.5**	**3**	**3**	**6***
**>54**	**2**	**4**	**2**	**6**	**4**	**12***

***Persons on the high Rif arm will be dispensed the 300mg capsules to reduce pill burden.**

Treatment will be administered during patients' scheduled visits and this will be monitored by trained community-based volunteers and health workers. Skin graft will be provided for all patients with ulcers that enlarge after 8 weeks of antibiotics treatment upon recommendation from the Technical Expert Panel.

4.3 OTHER MEDICATIONS

4.3.1 MEDICATION(S)/TREATMENT(S) PERMITTED BEFORE AND/OR DURING THE TRIAL

All treatments which are not specified as being contraindicated may be administered as required during the study period. This includes routine analgesics for pain and malaria should be treated according to local practice, with the exception of Fansidar (sulfadoxine/pyrimethamine combination) which should be avoided if possible.

All concomitant medication taken during the study will be recorded in CRF with trade name and generic name, route or formulation, dosing scheme, the indication and start and stop dates of administration.

Since Rifampicin induces metabolism of anti-hypertensive drugs and decreases their potency, hypertensive participants on anti-hypertensive drugs will have their blood pressure closely monitored to allow for appropriate decision making. As part of the trial team, an experienced Consultant Physician with extensive experience in the management of high blood pressure will be available to make decisions regarding adjustment to doses of antihypertensives if need be.

4.3.2 Medication(s)/treatment(s) not permitted before and/or during the trial

All treatments which are specified as being contraindicated will be avoided. If therapy of these drugs is required for any reason, then the patient will be withdrawn from further anti-mycobacterial drug treatment and the appropriate treatment as determined by local treatment guidelines and protocols, and as prescribed by the attending physician will be offered.

4.3.3 Contraindications to rifampicin

These medicines may increase the chance of liver damage if taken with Rifampicin:

- Acetaminophen (with long-term, high-dose use)
- Amiodarone
- Anabolic steroids
- Androgens
- Antithyroid agents
- Carbamazepine
- Carmustine
- Chloroquine
- Dantrolene
- Daunorubicin
- Disulfiram
- Divalproex
- Etretinate
- Gold salts
- Hydroxychloroquine
- Isoniazid
- Mercaptopurine
- Methotrexate
- Methyldopa
- Naltrexone (with long-term, high-dose use)
- Phenothiazines
- Plicamycin
- Valproic acid

Rifampicin may decrease the effects of these medicines:

- Anticoagulants
- Aminophylline
- Amprenavir
- Antidiabetics
- oral Chloramphenicol
- Corticosteroids
- Delavirdine
- Digitalis glycosides
- Disopyramide
- Efavirenz
- Fluconazole
- Indinavir
- Itraconazole
- Ketoconazole
- Methadone
- Mexiletine
- Nelfinavir
- Nevirapine
- Oxtriphylline
- Quinidine
- Ritonavir
- Saquinavir
- Theophylline
- Tocainide
- Verapamil

Rifampicin may decrease the effects of these medicines. These medicines may also increase the chance of liver damage if taken with rifampicin:

- Estramustine
- Estrogens
- Oral contraceptives
- Phenytoin

4.3.4 Contraindications to Clarythromycin

- Acenocoumarol
- Alfentanil
- Astemizole
- Carbamazepine
- Cisapride
- Clindamycine
- Cyclosporine
- Digoxin
- Disopyramide
- Midazolam
- Omeprazole
- Phenytoin
- Pimozide
- Quinidine
- Rifabutin
- Ritonavir
- Statine medicines
- Tacrolimus

- Ergot alkaloid medicines and ergotamine derivates
- Fluconazole
- Hexobarbital
- Indinavir
- Lincomycine
- Terfenadine
- Theophylline
- Triazolam
- Warfarin
- Zidovudine

4.4 Participant Withdrawal criteria

4.4.1 Criteria for Withdrawal from trial treatment

i. Pregnancy in the course of the period of active treatment
ii. Development of allergy to the trial treatments

4.4.2 Criteria for Withdrawal from the trial

i. Withdrawal of consent for any reason at the request of the patient or the treating physician

Participant has the right to withdraw from the trial without giving any reasons, however, if they wish to give reasons, it will be accepted and documented. Patients withdrawing from the trial treatments will be switched to standard care (standard dose Rifampicin and Vaseline gauze) with routine local follow-up. Benefits from the study (medications, lesion management and wound care with standard materials) will continue even if a patient withdraws from the study. **Females who are detected to be pregnant after start of study medication will be withdrawn and followed up by an Obstetrics Gynecologist throughout the pregnancy to assess any effect on the pregnancy outcome. If a life baby results, the baby will be followed up by a pediatrician for two years.**

Data collected up to the point of withdrawal will be maintained in compliance with GCP to facilitate audit by the sponsor and regulatory bodies.

STUDY PROCEDURES

5.1 Sensitisation

The study team will participate in ongoing outreach community education and early detection activities in the districts and will organize meetings with opinion leaders in endemic areas in each district. These opinion leaders include health care workers, (i.e. village health workers, traditional birth attendants, traditional and herbal healers) as well as other important social leaders, (e.g. school teachers, chiefs and elders). The outline of the study protocol will be explained to them, and culturally sensitive issues will be addressed in detail. They will be informed on the nature of the study; the benefits and disadvantages involved; and/or schools to be approached. All willing individuals or parents / relatives / guardians / legal representatives (for study participants under 18 years) will be informed beforehand of the details and intentions of the study during the community education meetings and referred to the clinics.

ACTIONS TAKEN TO MINIMIZE RISKS OF CONTRACTING COVID-19

GUIDELINES FOR PATIENT RECRUITMENT AND OUTREACH PROGRAMS

In the event that an outreach needs to be organized, the following guidelines will be followed:

- Employing the door-to-door method, following public health guidelines on social distancing. This will involve the use of pictures (WHO pictorial guide for health workers) and other visual aids to enhance communication.
- In a gathering of more than 25 people, guidelines and social distancing protocols should be adhered to and the event be organized at a large space to accommodate everyone.
- In all outreach activities, wearing of nose masks and hand washing protocols must be adhered to according to the public health and the WHO guidelines.
- Other protocols for the conduct of outreach programs as stated in the study protocol section will be followed.

5.2 Screening and recruitment

There will be facility-based recruitment of patients who seek treatment for BU following due counselling and consent. Clinical diagnosis will be made by one experienced clinician (lead clinician at each site), who will also take the samples. Guidance on clinical diagnosis of BU is provided in WHO pictorial guide for health worker. These are normally done as part of routine care of all patients presenting as suspected BU. Patients who refuse to participate in the study or withdraw at any time will still receive all necessary care.

Patients will be provided with a full explanation of the nature, purpose and requirements of the study including information sheets and informed consent forms. They will be invited to

participate in a screening evaluation which will include a medical history, physical examination and wound assessment.

All females of the reproductive age will be screened for pregnancy using the Urine Pregnancy Test (UPT) kits and information on the last menstrual period. This will be done at weeks 0, 4, and 8. Females who are found pregnant at the point of screening will be excluded. Counselling will also be given to all females of reproductive age who are not pregnant at week 0 on the need for contraception (including barrier methods such as female and/or male condoms) and avoidance of pregnancy during the period of taking the study medication.

To recruit participants the following steps are required:

1. Detailed explanation of the study to participants (Informed Consent Procedures, ICH-GCP, ANNEX A and B) in the local language Twi or English.

2. Ensure the participant (or their parent/guardian) has a complete understanding of the study and its procedures. Make the participant aware of the benefits and the minimal risks. To ensure that the participant has had the chance to ask questions regarding their participation in the trial and has signed the Informed Consent Form (ICF) (ANNEX B).
3. Clinical examinations including:
   - Demography and medical history
   - Concurrent medications
   - Lesion assessment
   - **Diagnostic tests for *Mycobacterium ulcerans***

Each participant will be informed of the voluntary nature of the study and be encouraged to ask questions throughout the period of investigation.

COVID-19 GUIDELINES ON PATIENT RECRUITMENT AND INFORMED CONSENT PROCEDURE

- Before the participant gets to the clinic, hospital guidelines for visitors would be followed including hand washing and temperature checks. Before entering the premises of the hospital, participants will be expected to wear face masks. This will be provided to participants
- This will also be enforced at the BU clinic.
- **The study will provide veronica buckets, running water and soap to ensure that both researcher and the study participant will follow hand washing protocols at the clinic.**
- **Alcohol base hand sanitizers will also be available for both researcher and participant at the BU clinics.**
- At a private sitting place, a distance of about 2m will be created between the participant and the researcher for the explanation of the study to be done.
- We will ensure that both researcher conducting the consent procedure and the eligible participant will wear face mask covering their nose and mouth throughout the session.
- Witnesses who need to be present during the procedure will also sit at a distance not less than 2 m from both researcher and participant, however distance should not prevent clear

hearing of every communication going on. Time should be taken to explain every part of the study as written on the information leaflet.

- Wherever possible, where it may not interfere with the study protocols, telephone conversations can be arranged.

Treatment decisions for BU will be based on the PCR result as for all other patients in routine care. PCR is the current gold standard diagnostic test. Normal care of all patients with BU involves reporting for review at week 2, 4, 6, 8 and then monthly for 1 year following initiation of antibiotic treatment at the local BU clinic (normally at the district level hospital/health facility). This is necessary to monitor their response to therapy and recurrence of lesions. A window period of plus or minus 2 weeks will be allowed for study participants who miss their appointments for samples to be collected. Patients who miss their appointment are followed up by phone. To facilitate retention, transportation costs for patients will be paid for by the research study as in previous trials. Visits up to **week 8** will be used for data collection for the primary analysis of the study. Visits after week 20 will take place as part of patients' routine care.

Based on the number of BU patients seen across the selected districts we expect to recruit 8 participants a month leading to a total recruitment time of 14 months.

COVID-19 Guidelines on Patient Follow- ups

Normal care of all patients with Buruli ulcer, involves reporting for review at week 2, 4, 6, 8 and monthly for 1 year following initiation of antibiotic treatment to monitor response to therapy.

The following guidelines will however be followed in the period of COVID-19

- Patients whose lesions heal within the first 8 weeks of antibiotic treatment shall be required to come for review on monthly basis. Drugs will be given to cover the period before hospital scheduled review and WHO standard trained village volunteers will be deployed to monitor drug adherence biweekly.
- All others who will be maintained on the two weekly review will follow all COVID -19 guidelines as stated.

5.2.1 Randomisation and Blinding

Patients will be randomised at the point of enrolment. It will not be possible to blind participants or clinicians to the treatment the participant receives. **The process of the randomization will be such that, participants are given randomly generated allocations within sealed envelopes. Once a patient has consented to participate in the trial and has satisfied all the inclusion criteria, an envelope corresponding to their study specific number will be opened and the patient is then offered the allocated treatment regimen. A skilled blinded wound assessor will be tasked to evaluate the wound images captured by the Silhouette camera for healing to avoid bias and maximize validity of results.**

5.2.2 Initial Sample collection and confirmation

Two swabs will be taken from the undermined edges of the ulcerated lesions or 2 FNAs from non-ulcerated lesions at diagnosis for MU PCR and 16S rRNA to confirm the diagnosis of BU.

GUIDELINES ON CLINICAL DIAGNOSIS, SAMPLE COLLECTION AND CONFIRMATION.

The following guidelines may be followed.

- For every eligible study participant to be examined, there should be no more than two health workers / researchers in the examination room at a given time.
- Both health workers and the study participant must wear face masks and in the case the study participant does not have, the researchers must provide one.
- All protocols for proper hand washing using soap and running water/ use of alcohol-based sanitizers should be followed. This should be done before attending to the participant and immediately after attending to the participant.
- All other aseptic techniques required according to all health guidelines must be adhered to especially during the sample collection step as was stated in the study protocol
- As much as possible social distancing should be ensured at the waiting area to be achieved by modified seat arrangements.
- Participants expressing other signs (Mostly COVID-19 related) should be referred to the hospital COVID-19 management team for further investigations.
- Other procedures for sample collection and laboratory confirmation will be followed as stated in the study protocol.

Detection of MU by PCR

Swabs or FNA will be processed for PCR IS2404 using established methods in laboratory that currently provide PCR confirmation for routine BU cases. Samples will be processed at KCCR in Kumasi and NMIMR in Accra. PCR results will be provided free of charge to all patients who participate or do not participate. The principal investigator (RP) is the responsible person for overseeing the Kumasi Centre for Collaborative Research diagnosis for BU under the WHO Global Network of Laboratories for Confirming *Mycobacterium ulcerans* disease (Buruli ulcer) and will facilitate this[18].

MU culture

Swabs or FNA will be inoculated onto Lowenstein Jensen slopes for culture and microscopy will be carried out as described previously[18].

Combined 16S rRNA reverse transcriptase/IS2404 real-time qPCR assay

For the combined 16S rRNA reverse transcriptase/IS2404 real-time qPCR assay to assess viability of MU, swabs and FNAs will be stabilised in 500ul of RNA protect (Qiagen) and subjected to combined DNA/RNA extraction by the All-Prep DNA/RNA extraction kit (Qiagen). RNA will be reverse transcribed to cDNA. DNA and cDNA will be subjected to IS2404 qPCR and 16S rRNA real time PCR respectively[9].

5.3 Data Collection:

5.3.1 Clinical Data Collection

Data will be collected at enrolment and at follow-up at study time points. Clinical and treatment data will be collected using standard CRFs based on the WHO BU 01 form[19]. Photos of the lesion will be taken for documentation purposes, consistent with routine care.

5.3.1.1 Wound size

Wound diameter and area will be traced on acetate sheets, recorded by digital photography and measured by computer planimetry using a Silhouette® Wound Imaging System at each clinical visit. The Silhouette camera captures an image of the wound, a tracing of the wound boundary is generated and the wound dimensions are automatically calculated. These measurements will enable calculation of healing rate at week 4 and predicted healing time in relation to lesion size and type. The mean diameter, area, depth and volume of the wound will be measured. The same measurements will be made for non-ulcerated lesions (nodules and plaques). Assessment will be blinded/ wound images evaluated for healing / measured by a skilled blinded assessor

5.3.1.2 MU viability assessment

Samples will be taken before and during treatment to assess persistence of viable mycobacteria. Swabs will be taken from the **undermined edges of ulcerated lesions or FNA samples from the centre of non-ulcerative (nodule, plaque and oedema) lesions** at weeks 2, 4, 8, 12 and 16 (if lesions are not healed) for the combined MU 16S rRNA reverse transcriptase/IS2404 real-time qPCR[9,10].

5.3.1.3 Secondary Infection

Wounds will be assessed clinically for secondary infection at each time point. In the context of new purulent discharge, a single swab shall be taken for microbiological assessment at **KCCR and NMIMR Laboratories.**

Bacterial culture

Swabs will be plated onto agar and cultured for the identification of potential secondarily infecting pathogens. Organisms will be identified and antibiotic sensitivity testing performed using standard microbiological protocols.

5.3.2 Economic and quality of life data collection

Data on health-related quality of life will be collected at enrolment and at each follow-up visit in appropriate versions of the EQ-5D (EQ-5D-5L or EQ-5D-Y for children), a simple, 5-question instrument validated for Ghana, which allows for estimation of quality-adjusted life-years (QALYs)[20]. In addition, brief questions will assess the patient's feelings about social perceptions of their BU and experience of pain or itch to assign a disability-adjusted life-year (DALY) weight to the patient's health state.

Additional data on socio-economic status, the costs of living with BU, and the resources and costs associated with seeking and providing treatment for BU will also be collected. These data will be collected from BU patients and their families via additional questions at planned clinical contacts; review of patients' clinical records; observation of patients' contacts with the healthcare system; review of facility records; review of research study budget and activity records; and interviews with clinicians and community volunteers. Data on salary scales and budgets associated with BU treatment will also be sought from relevant sources.

Economic and quality of life data collection tools will be piloted in a sample of <10 patients receiving usual care for BU prior to enrolment of patients in the trial. Tools will be refined iteratively through the piloting process to ensure that they are well understood, acceptable, and feasible.

5.4 Adverse Events:

Term	Definition
**Adverse Event (AE)**	Any untoward medical occurrence in a participant to whom a medicinal product has been administered, including occurrences which are not necessarily caused by or related to that product.<br><br>An AE can therefore be any unfavourable and unintended sign (including an abnormal laboratory finding), symptom, or disease temporally associated with the use of an investigational medicinal product (IMP), whether or not considered related to the IMP.
**Adverse Reaction (AR)**	Any untoward and unintended response in a participant to an investigational medicinal product which is related to any dose administered to that participant.<br><br>The phrase "response to an investigational medicinal product" means that a causal relationship between a trial medication and an AE is at least a reasonable possibility, i.e. the relationship cannot be ruled out.<br><br>All cases judged by either the reporting medically qualified professional or the Sponsor as having a reasonable suspected causal relationship to the trial medication qualify as adverse reactions.
**Serious Adverse Event (SAE)**	A serious adverse event is any untoward medical occurrence that:<br>• Results in death<br>• Is life-threatening<br>• Requires inpatient hospitalisation or prolongation of existing hospitalisation<br>• Results in persistent or significant disability/incapacity<br>• Consists of a congenital anomaly or birth defect<br><br>Other 'important medical events' may also be considered serious if they jeopardise the participant or require an intervention to prevent one of the above consequences.
**Serious Adverse Reaction (SAR)**	An adverse event that is both serious and, in the opinion of the reporting investigator, believed with reasonable probability to be due to one of the trial treatments, based on the information provided.
**Suspected Unexpected Serious Adverse Reaction**	A serious adverse reaction, the nature and severity of which is not consistent with the information about the medicinal product in question set out:

**(SUSAR)**	• In the case of a product with a marketing authorisation, in the summary of product characteristics (SmPC) for that product<br>• In the case of any other investigational medicinal product, in the investigator brochure (IB) relating to the trial in question.

5.4.1 Assessment procedures

Most adverse events and adverse drug reactions that occur in this study, whether they are serious or not, will be expected treatment-related side effects due to the drugs used in this study. The assignment of causality should be made by the investigator responsible for the care of the participant using the definitions in the table below.

If any doubt about the causality exists the local investigator should inform the study coordination centre who will notify the Chief Investigators. The pharmaceutical companies and/or other clinicians may be asked to advice in some cases.

In the case of discrepant views on causality between the investigator and others, all parties will discuss the case. In the event that no agreement is made, both points of view are to be reported.

Relationship	Description
**Unrelated**	There is no evidence of any causal relationship
**Unlikely**	There is little evidence to suggest there is a causal relationship (e.g. the event did not occur within a reasonable time after administration of the trial medication). There is another reasonable explanation for the event (e.g. the participant's clinical condition, other concomitant treatment).
**Possible**	There is some evidence to suggest a causal relationship (e.g. because the event occurs within a reasonable time after administration of the trial medication). However, the influence of other factors may have contributed to the event (e.g. the participant's clinical condition, other concomitant treatments).
**Probable**	There is evidence to suggest a causal relationship and the influence of other factors is unlikely.
**Definitely**	There is clear evidence to suggest a causal relationship and other possible contributing factors can be ruled out.
**Not assessable**	There is insufficient or incomplete evidence to make a clinical judgement of the causal relationship.

5.5 Reporting

An independent Data Safety and Monitoring Board (DSMB) will be established to review, in strict confidence, data from the trial approximately every six months. The Chair of the DSMB may also request additional meeting/analyses. One scheduled interim analysis will be performed by an independent statistician once approximately half of the total sample size is achieved. In the light of these data and other evidence from relevant studies, the DSMB will inform the Trial Steering Committee**. This may inform the stopping or discontinuation of the study if:**

i. **they have a major ethical or safety concern.**
ii. **interim analysis reveals clear benefits in favor of one study arm over the other.**
iii. **there is proof that the data indicate that any part of the protocol under investigation is either clearly indicated or clearly contra-indicated either for all patients or a particular subgroup of patients, guided by the stringent Peto-Haybittle rule (Haybittle JL: Repeated assessment of results in clinical trials of cancer treatment. Br J Radiol 1971, 44(526):793-797). The Peto Haybittle rule does not require any adjustment to alpha at the final analysis as it is based on a very stringent probability of equal to, or less than 0.001.**
iv. **it is evident that no clear outcome will be obtained with the current trial design.**
v. **a female participant in her reproductive age falls pregnant whiles taking the study medication.**

Depending on the nature of the event the reporting procedures below should be followed. Any questions concerning adverse event reporting should be directed to the study coordination centre in the first instance.

5.5.1 *SERIOUS ADVERSE REACTIONS (SARS)/SERIOUS ADVERSE EVENTS (SAES)*

Serious Adverse Events (SAEs) and Serious Adverse Reactions (SARs) should be reported to the study coordination centre within 24 hours of the local site being made aware of the event.

An SAE form should be completed and submitted to the study coordination centre with as much detail of the event that is available at that time. If awaiting further details, a follow up SAE report should be submitted promptly upon receipt of any outstanding information. The PI will record the event with an assessment of seriousness, causality and expectedness.
Once AEs and/or SAEs are identified, participants will be offered appropriate treatment within the health facilities and followed up or referred to a higher level of care if required. One of the study physicians; a consultant physician at KATH will be available to coordinate and manage all AEs and/or SAEs.
Any events relating to a pre-existing condition or any planned hospitalisations for elective treatment of a pre-existing condition do not need reporting as SAEs.

5.5.2 *SUSARS*

All SAEs assigned by the PI or delegate as both suspected to be related to IMP-treatment and unexpected will be classified as SUSARs and will be subject to expedited reporting to the Regulatory Authority. The Sponsor (or delegate) will inform the relevant bodies of SUSARs within the required expedited reporting timescales.

In the case of a suspected, unexpected, serious adverse reactions (SUSAR), the staff at the site should:

1. Contact the study coordination centre immediately by phone or email to inform them of the event.
2. Submit a completed SAE form (signed and dated) within 24 hours, together with relevant treatment forms and anonymised copies of all relevant investigations.
3. Submit any additional information promptly upon request.

5.6 Urgent safety reporting

It may be necessary to take appropriate urgent safety measures to protect participants from any immediate hazard to their health and safety. These measures will be taken immediately. It is not necessary to wait for ethical approval before implementing the safety measure; however, both ethical review committees and the Sponsor will be informed in writing within three working days by submitting a substantial amendment.

5.7 Quality control and validation

Standard operating procedures will be in place for clinical diagnosis, sample collection, transport and storage as well as all trial related activities. Doctors and nurses will cross check patient clinical and laboratory data. Data entry staff will enter data by double entry. Staff involved in the study will have Good Clinical Practice and Good Laboratory practice training.

6 ANALYSIS & STATISTICAL CONSIDERATIONS

6.1 General design

This is an individually randomised open-label study of high-dose Rifampicin and DACC dressing, compared to current standard dose Rifampicin and DACC dressing.

Assessment will be blinded and wound images evaluated for healing / measured by a skilled masked assessor.

6.2 Sample size justification

Previous studies[9] have demonstrated a mean time to clearance of viable mycobacterium with standard dose Rifampicin (standard care) as 9 weeks, with a standard deviation of 4 weeks. Assuming a lower standard deviation of 3 weeks for high-dose Rifampicin (intervention), 100 participants (randomised 1:1) would enable us to detect a reduction of 2 weeks in mean time to RNA clearance with 80% power and 5% significance.

In addition, 100 participants would also allow us to see an increase of approximately 27% with healed wounds at 20 weeks in the high-dose Rifampicin arm. This is based on a conservative assumption that 50% of wounds would be healed at 20 weeks with DACC dressings and the standard dose Rifampicin[9].

Assuming a loss to follow-up rate of 10%, we therefore plan to recruit 112 participants to ensure that at least 100 participants are followed up for a minimum of 20 weeks.

The random allocation sequence will be computer-generated at LSHTM using block randomization stratified by site and BU stage and placed in sealed opaque envelopes. This will be done in a secure manner to avoid any possibility of viewing the allocation sequence. The participating clinician will be given randomly generated treatment allocations within sealed opaque envelopes and once a patient has consented to participate in the trial and has satisfied all the inclusion criteria, an envelope corresponding to their study specific number will be opened and the patient is then offered the allocated treatment regimen. A skilled blinded wound assessor will be tasked to evaluate the wound images captured by the Silhouette camera for healing to avoid bias and maximize validity of results.

Randomization codes linking allocation to study number will be held by the statistician until the database is unlocked. The local monitor and chair of the DSMB can request for randomization codes from the statistician in case of an individual safety concern.

6.3 Statistical methods

Statistical analysis of outcomes will be carried out blind to treatment allocation.

Screening, enrolment, reasons for non-enrolment, randomization and loss to follow-up will be detailed in a CONSORT flowchart.

The primary and secondary efficacy outcome analysis will be carried out using a modified intention-to-treat (ITT) population of individuals who were PCR positive for BU at baseline. An analysis on a per-protocol population will also be carried out.

Analysis will follow a pre-specified analysis plan approved by the senior statistician, Chief Investigator and the DSMB prior to unblinding the study database. Every effort will be made to obtain outcome measures on all participants. Demographic, clinical and other characteristics at trial entry will be tabulated. No significance tests will be performed to test for differences at baseline. Descriptive statistics for continuous variables will include the mean, standard deviation, median, range and the number of observations. Categorical variables will be presented as numbers and percentages.

For the primary outcome of mean time to clearance of viable mycobacterium, we will use regression models with a Gaussian link function to estimate the mean difference between the two arms. This will be reported with a 95% confidence interval.

For secondary outcomes, appropriate models will be used to examine the effect of the intervention. Appropriate measures of effects will be reported with 95% confidence intervals.

If there is evidence of non-normality in the continuous outcomes, then non-parametric bootstrapping will be used to estimate bias-corrected 95% confidence intervals.

Every effort will be made to obtain required data at each scheduled evaluation from all participants who have been randomised. The data coordinating centre will be responsible for logging all data as they arrive and contacting the centres about any missing data. Missing data will be chased until received, confirmed as not available, or the trial is at analysis. Data quality, follow up and trial monitoring will be facilitated through the development of a trial specific database, including validation, verification, monitoring and compliance reports and follow up report functionalities. Therefore, only a small amount of missing data is expected and it is not thought likely that it will have to be accounted for in any analysis.
However, we would consider using inverse probability weighting or multiple imputation if missing data were larger than expected and/or there was differential attrition between the trial arms. We would also attempt to ensure that the reason for the differential attrition was fully understood.

The primary analysis will be an unadjusted analysis.

An adjusted analysis will be carried out, adjusted for the stratification factor site with any other adjustment factors pre-specified in the statistical analysis plan. Additional exploratory analyses will control for any baseline measures that appear to be imbalanced between arms. All subgroup analyses will be specified a priori in the statistical analysis plan and carried out using formal tests for interaction included in the statistical models and assessed for statistical significance using Likelihood ratio tests.

6.4 Populations to be analysed

Analyses will be carried out using two different types of datasets.

i) The modified intention-to-treat datasets: Data will be analysed based on all patients who were PCR positive for BU at baseline, according to the random allocation irrespective of whether the intervention was or was not entirely or partly taken up.

ii) The per-protocol datasets: As it is possible that patients may not receive the intervention, the intention-to-treat analysis might underestimate the potential effectiveness of intervention. A per-protocol analysis will therefore be carried out in addition to the intention-to-treat analysis. The per-protocol datasets will include data pertaining to all outcomes, provided that all the following criteria apply: (i) completion of the course of antibiotic treatment; (ii) undergoing advanced wound care therapy; (iii) no major protocol violation with regard to inclusion/exclusion criteria; and (iv) no prohibited medications taken during the study treatment period.

6.5 Efficacy Endpoints

Determination of the clinical efficacy endpoints will be measured as follows:

Primary Endpoint:

- Time to clearance of viable mycobacterium. **This will be evaluated at week 8.**

Secondary Endpoints:

- Proportion of wounds healed at 20 weeks
- Recurrence of BU within study period
- Incidence of paradoxical reaction
- Incidence of secondary infection
- Cost and Cost-effectiveness

Indicators for each outcome are given below

**Outcome**	**Indicator**
Primary outcomes	
Time to clearance of viable mycobacterium	• Proportion of patients with no viable organisms, as assessed by Combined 16S rRNA reverse transcriptase/IS2404 real-time qPCR assay, detectable at weeks 2, 4 and 8 during antibiotic treatment.
Secondary outcomes	
Proportion pf patients with healed wound at 20 weeks	• The number patients achieving complete healing of lesions at 20 weeks.<br>• Complete healing is defined as re-epithelialisation of ulcerated area with stable scar formation during a three-month observation period.
Recurrence of BU	• Proportion of patients with recurrence in each treatment arm.<br>**Recurrence** refers to a new lesion detected within 12 months of treatment which is not considered a paradoxical reaction.

Outcome	Indicator
	It is mainly characterised by the presence of viable organisms detected by culture or 16S rRNA assay after completion of antibiotic treatment and complete healing of the initial lesion.<br><br>Any new lesion thought to contain viable organisms will be assessed by a panel of experts blinded to the treatment allocation using available resources including laboratory results, photographic documents and notes from the patient files to decide if it represents a recurrence. This will however be reported as recurrence to be 'Proven', 'likely/ probable' or 'unlikely/rejected'
Incidence of paradoxical reaction	• Proportion of patients with paradoxical reaction in each treatment arm.<br><br>**Paradoxical reaction** is defined by the presence of one or both of the following features: (i) an initial improvement in the clinical appearance of an *M. ulcerans* lesion upon antibiotic treatment, followed by deterioration of the lesion or its surrounding tissues or the appearance of a new lesion(s); and (ii) examination of excised tissue from the clinical lesion showing evidence of an intense inflammatory reaction consistent with a paradoxical reaction
Incidence of secondary infection	• The proportion of patients who experience a secondary infection.<br><br>**A secondary infection** is defined as the occurrence of new purulent discharge within a BU lesion accompanied by microbiological evidence of infection.
Cost and Cost-effectiveness	• See Section 6.6 Economic Evaluation

6.6 Economic Evaluation

6.6.1. Cost and budget impact analysis

We will triangulate data collected in the trial to generate best estimates of the financial and economic costs to providers and to households of experiencing and treating BU. We will estimate costs as incurred in the trial and, separately, as would be expected to be incurred in "real-world" treatment. For example, while transport costs for patients (and their caregivers, where appropriate) to travel to receive treatment will be paid for by the trial budget, outside of the trial, patients' households would normally need to cover these costs themselves; we will

38

show these costs as a provider cost in the “within-trial” analysis, and as a cost to households in our estimates of “real-world” costs.

We will examine budget impact by estimating the incremental financial costs (or cost savings) per household, and to relevant provider budgets, which may include health facilities, third party payers (insurance), national budgets for neglected tropical disease control, and donor budgets. We will compare cost burden with existing budgets, where relevant, and engage with policy makers to understand affordability.

At the household level, all direct costs related to the treatment of BU will be computed at the household level. This will then be compared to the household’s wellbeing indicator (income or expenditure). Households whole budget share of BU expenditure exceeds a pre-determined threshold will be considered catastrophic. This threshold will be in line with standard practice in the literature (Wagstaff, 2018). We will also assess whether BU related spending pushes the household into or further into poverty. This will be done by simply comparing poverty levels at the household before and after incurring BU spending (Wagstaff and Doorslaer, 2003).

From the perspective of the facility, the budget impact will be estimated as the incremental financial burden of the new treatment regimes. In practical terms, this will simply be the cost of the standard treatment regime minus the cost of the new treatment regime (Dee et al. 2012). To do this, we will compute the cost of all resources used in treatment across the three treatment arms. As mentioned earlier, this additional cost (savings) from the new treatment regime will be analysed in relation to provider budgets and from the perspective of different stakeholders. This is particular important when budget financing is from multiple sources. To ensure consistency, this analysis will follow standard guidelines are suggested in Sean et al (2014).

Economic cost estimates will be used to examine efficiency in our cost-effectiveness analysis, described below.

6.6.2. Analysis of effectiveness

In addition to the primary and secondary trial outcomes, we will also estimate the difference in effectiveness of high-dose Rifampicin and DACC dressing in terms of DALYs averted and quality-adjusted life-years (QALYs) gained. DALYs and QALYs both combine measures of length and quality of life into a single metric, which are important in allowing the results of economic evaluations to be compared across different health areas.

To estimate DALYs, we will use patient and clinician responses to assign an appropriate DALY weight for each patient at each time point. We will then combine DALY weights with an estimate of the duration over which the health state was experienced to identify the DALYs lost by each patient (relative to perfect health) over the 12-month duration of the trial. To estimate QALYs, we will combine results of the EQ-5D with an appropriate valuation set to estimate each patient’s QALY weight at each time point. We will then combine the QALY weight with an estimate of the duration over which that health state was experienced to estimate the QALYs gained for each patient (relative to not being alive) over the 12-month duration of the trial. The QALYs gained and DALYs lost by each patient (relative to a theoretical maximum of 1 over 1 year) will therefore be

assessed as the "area under the curve" as delineated by the QALY and DALY weights at each time point.

Mean differences in DALYs lost and QALYs gained between treatment strategies will be estimated using appropriate regression methods, controlling for baseline differences in DALY and QALY weights[21]. Evidence for long-term differences in outcomes will be assessed by comparing mean differences in DALY and QALY weights assigned at the 12-month trial endpoint, controlling for baseline differences, for patients with 12 months' follow up. For patients with < 12 months' follow up available, appropriate imputation methods will be used.

6.6.3. Cost-effectiveness analysis

If the high dose Rifampicin-DACC dressing strategy is found to be less effective, it will not be recommended for adoption, cost-effectiveness analysis will not be conducted, and the economic data collected will be used to inform alternative analyses of the economics of BU treatment. If the high dose Rifampicin-DACC dressing strategy is found to be both more effective and less costly than current strategy over the 12-month time horizon of the trial, the high dose Rifampicin-DACC dressing strategy will be considered "dominant", i.e. the economically superior strategy. We assume that if the high dose Rifampicin-DACC strategy is dominant over 12 months, it would remain the dominant option over a lifetime horizon.

If the high dose Rifampicin-DACC dressing strategy is found to be both more effective and more costly than alternative strategies, we will estimate the incremental cost-effectiveness ratio (ICER), which will allow us to compare the relative efficiency of investing in the high dose Rifampicin-DACC dressing strategy with alternative possible uses of the additional resources. The ICER is calculated as the difference in mean costs between the intervention groups divided by the difference in mean effects between groups. Findings will be expressed as the incremental cost per QALY gained and the incremental cost per DALY averted. Deterministic and probabilistic sensitivity analyses will be conducted to assess the robustness of results to uncertainty and heterogeneity.

If there is evidence for long-term (beyond 12-month) differences in the mean DALYs or QALYs associated with the interventions, we will model costs and effects over a lifetime horizon and base our ICER estimates on these long-term estimates. Model structure and parameters will be informed by existing literature and by consultation with experts within the trial team and wider advisory committee, including patient representatives where appropriate. Otherwise, we will consider the study period sufficient to capture all relevant differences in costs and effects between the high dose Rifampicin-DACC and alternative strategies.

6.6.4 Equity analysis

In accordance with reference case specifications, if the HR+DACC dressing strategy is found to be more effective than alternative strategies, we will explore the equity of switching to HR+DACC strategy. We will do this by examining variation in incremental costs and benefits to households across socio-demographic groups, using data collected from each patient.

7.0 DATA MANAGEMENT AND MONITORING

7.1 Source documentation

Complete source documentation (e.g. laboratory test reports, medical records) is required for each participant for the entire duration of the study. Case Report Forms (CRFs) will be used to collect data for participants enrolled in the study. Data collected in the CRFs should be consistent with the source documents; any discrepancies should be explained using file notes.

7.2 Study documentation and data safety

The study databases will be password protected and access to password will be authorised by the Principal Investigators. Electronic data files will be stored on operated and dedicated servers at KCCR/LSHTM. Analyses of the data will be performed on copies of the original data files ensuring raw data accessibility at all times. Regular backups will be carried out ensuring protection against data loss.

Privacy of the study participants will be maintained by assigning study participants a unique study identification number. All data, samples and laboratory results will be recorded and analysed with only this unique identification number and no personal identifiers. All information collected, including names, specific addresses, etc. that could identify participating participants will be kept confidential, separate from the rest of the trial data and available only to the investigators and authorised study personnel. We will keep confidential any information related to this study and all data and records generated in the course of conducting the study, and will not use the information, data, or records for any purpose other than those specified in this study protocol.

All samples from BU patients will be anonymised. At recruitment, a unique code will be assigned to each participant and an identification card will be issued. The link between name and identification code will be accessible only to the Study Manager. No name or identifier will be used in any publication or reports from this study.

Diagnostic confirmation results will be made available to the treating physician in the various hospitals. This will ensure that patients benefit from optimal treatment.

7.3 Data entry and storage

All written forms (i.e. consent and CRFs) will be stored in the KCCR/KNUST and NMIMR laboratories in Ghana. All forms will be labelled and filed in lockable filing cabinets with the Study Protocol Name, Principal Investigators' names and collection dates. Keys will be kept with the principal investigators or the persons responsible for data management. Study-related documents will be maintained by the investigator for a period of 10 years following completion of study. It is the duty of the sponsor to inform the investigator when documents can be discarded**.** The electronic database will be stored for 25 years by the Principal Investigators.

7.4 Study monitoring

An external GCP monitor will be appointed to monitor all aspects of the study, with respect to current GCP, to ensure compliance with government regulations. Prior to the start of the study, the investigator will be informed of the frequency of monitoring visits and will be given reasonable notification before each visit by other designated monitoring bodies.

The objectives of a monitoring visit will be to ensure:
1) prompt reporting of any harm resulting from invasive procedures during the study
2) availability of the signed ICF, ANNEX B
3) adherence to the GCP protocols
4) accuracy and completeness of the CRFs with source data.

During the monitoring visit, the investigator (and/or designee) and other study personnel should be available to discuss the study and any relating issues. Furthermore, all official study documentation must be available for review at all times by the monitor and all regulatory bodies including the FDA during GCP inspections.

8.0 RESULTS

Study results, once available, will be explained to participants in lay terms, on request. The results will be presented to the National Buruli ulcer control programme at the national Steering committee meeting and to local communities. Results will be provided to the Ethics Committee, the Regulatory bodies on request. Results will additionally be disseminated by publication in open access peer reviewed journals and presented at the annual WHO meeting on Buruli ulcer.

9.0 HUMAN RIGHTS PROTECTION

9.1 Research Ethics Committee

The Principal Investigator will be responsible for obtaining ethical approval. Before the start of the study, appropriate documents (including Study Protocol, Informed Consent Form and Patient Information Sheet, (ANNEX B) will be submitted for ethical approval in both Ghana and at LSHTM. A copy of the study approval (including a copy of all annexes) will be maintained in the trial master file and a copy will be supplied to the sponsor. The investigator will document study progress, in the form of an annual report, which will be submitted to the Ethics Committee, Ghana FDA and NIHR.

9.2 Informed Consent Form (ICF)

ICFs contain information on the purpose of research, types and amounts of clinical samples collected and their intended use, frequency and time intervals of specimen collection, a description of risks and benefits, statements on confidentiality of samples and personal data, the voluntariness of participation, and the right to withdraw from the study without any consequences regarding medical care. In view of illiteracy among rural African populations, thumb prints are foreseen as alternative to signatures. Informed (written) consent will be asked, both from the potential study participant as well as the parents / relatives / guardians / legal representatives (for study participants under 18 years). Assent shall be obtained from study participants between 13 and 17 years.

ACTIONS TAKEN TO MINIMIZE RISKS OF CONTRACTING COVID-19

In addition to the general study information to be given to the eligible study participants to allow them to make an informed decision to participate in the study, they will be given an additional information on COVID-19 pandemic. This information will be included in the Participant information leaflet and will contain basic information on the virus including public health guidelines for safety measures during COVID-19 pandemic.

Specific education will be given on hand washing procedures, social distancing and the importance of nose masks following the public health guidelines

Autonomy/coercion: Patient participation in this study will be voluntary. Participants will not be under any obligation to participate. Participants will be given enough time to consider participation. They will be given adequate time to discuss with family members, elders and others whom they choose. Even after agreeing to participate, participants will be allowed to withdraw from the study at any time for any reason. Participants will still be provided with usual/standard care if they decided to withdraw without any negative consequences.

Gender issues: There is no male or female pre-ponderance with Buruli ulcer. The research team consists of both female and male investigators. As caregivers of children are likely to be women the medical team will liaise with the health staff in the study areas to address issues that will affect their attendance to clinics and participation.

9.3 Benefits

The study will ensure that costs of tests and results of diagnostic confirmation by PCR are covered for patients that agree to participate and those that do not agree to participate to help establish the diagnosis for patients. Eventually the results of this study will be of benefit to future patients. Enhanced active case-finding activities will facilitate the patients' access to healthcare, including diagnostics and treatment. Local public health workers will benefit from training provided as part of the research programme.

9.4 Risks

Sample collection for the study is in line with standard diagnostic procedures carried out as part of routine patient care. Samples for diagnosis will be collected either via a swab or fine needle aspiration. Fine needle aspirates may cause minor pain. Only trained and experienced nurses and doctors will take samples. Procedures will be performed under sterile conditions to minimize the risk of infection for the patients. Any additional medical or nursing care will be delivered free of charge as is done in the routine care of all other patients with Buruli ulcer.

Data from several studies indicates that adverse events are no more common with high dose rifampicin than with standard dose.

Dressings may cause itch from the adhesive. When this occurs, they are advised to report to the doctor immediately as well as any other abnormal observations or ill feeling be reported whether related to the study or not.

9.5 Sample and data ownerships:

All biological samples obtained from participants for this study will be analysed within the lifetime of the project which is 5 years. Permission will be sought from the ethics committee and Ghana FDA should there be any unanticipated delay. All data obtained for the purpose of research will be managed by the investigators and not distributed. Test results for PCR will however be given to the patient and doctor responsible for the participant for optimal care. KCCR Kumasi and the Noguchi Memorial Institute for Medical Research (NMIMR) are both national Reference laboratories. Only KCCR and NMIMR laboratories will be involved in this study.

9.6 Compensation

Transport to clinic is not routinely paid for patients with Buruli ulcer outside of studies. In order to maximise retention within the proposed trial patients' transportation costs (a minimum of GH¢ 10.00) to routine clinics will be paid as part of the study. However, participants who will incur extra costs due to longer distances will be reimbursed accordingly. Standard PCR diagnostic tests done at the laboratory to confirm if the disease is Buruli ulcer or not will be paid for by this research study. This provision will also cover those that do not agree to participate. Any ill effect requiring additional medical or nursing care will be delivered free of charge.

9.7 Study registration

This trial will be registered at Pan African Clinical Trials Registry (PACTR) to obtain the Pan African Clinical Trials Registered Number (PACTRN), as required by the International Committee of Medical Journal Editors (ICMJE).

9.8 Declaration of interests

The principal investigators together with the study team wish to state that they have no conflicts of interest in the trial proposed.

9.9 Audits and Inspections

The study may be subject audit by the London School of Hygiene & Tropical Medicine under their remit as sponsor and other regulatory bodies to ensure adherence to GCP.

9.10 Indemnity

London School of Hygiene & Tropical Medicine holds Public Liability ("negligent harm") and Clinical Trial ("non-negligent harm") insurance policies that apply to this trial.

9.11 Quality Control and Validation

Standard operating procedures will be in place for clinical diagnosis, sample collection, transport and storage as well as all trial related activities. Doctors and nurses will cross check patient clinical and laboratory data. Data entry from written CRFs to the study database will use double entry. Staff involved in the study will have Good Clinical Practice training and follow Good Laboratory practice principles.

References

1 Amofah G, Bonsu F, Tetteh C, *et al.* Buruli Ulcer in Ghana: Results of a National Case Search. *Emerg Infect Dis* 2002; **8**: 167–70.

2 Vincent QB, Ardant M-F, Adeye A, *et al.* Clinical epidemiology of laboratory-confirmed Buruli ulcer in Benin: a cohort study. *Lancet Glob Health* 2014; **2**: e422–30.

3 Ackumey MM, Gyapong M, Pappoe M, Kwakye-Maclean C, Weiss MG. Illness meanings and experiences for pre-ulcer and ulcer conditions of Buruli ulcer in the Ga-West and Ga-South Municipalities of Ghana. *BMC Public Health* 2012; **12**: 264.

4 Grietens KP, Boock AU, Peeters H, Hausmann-Muela S, Toomer E, Ribera JM. "It Is Me Who Endures but My Family That Suffers": Social Isolation as a Consequence of the Household Cost Burden of Buruli Ulcer Free of Charge Hospital Treatment. *PLoS Negl Trop Dis* 2008; **2**: e321.

5 Barogui YT, Klis S, Bankolé HS, *et al.* Towards Rational Use of Antibiotics for Suspected Secondary Infections in Buruli Ulcer Patients. *PLoS Negl Trop Dis* 2013; **7**. DOI:10.1371/journal.pntd.0002010.

6 Demangel C, High S. Sec61 blockade by mycolactone: A central mechanism in Buruli ulcer disease. *Biol Cell* 2018; **110**: 237–48.

7 Sarfo FS, Phillips R, Wansbrough-Jones M, Simmonds RE. Recent advances: role of mycolactone in the pathogenesis and monitoring of Mycobacterium ulcerans infection/Buruli ulcer disease. *Cell Microbiol* 2016; **18**: 17–29.

8 Phillips RO, Robert J, Abass KM, *et al.* Rifampicin and clarithromycin (extended release) versus rifampicin and streptomycin for limited Buruli ulcer lesions: a randomised, open-label, non-inferiority phase 3 trial. *The Lancet* 2020; **0**. DOI:10.1016/S0140-6736(20)30047-7.

9 Sarpong-Duah M, Frimpong M, Beissner M, *et al.* Clearance of viable Mycobacterium ulcerans from Buruli ulcer lesions during antibiotic treatment as determined by combined 16S rRNA reverse transcriptase /IS 2404 qPCR assay. *PLoS Negl Trop Dis* 2017; **11**. DOI:10.1371/journal.pntd.0005695.

10 Frimpong M, Agbavor B, Duah MS, *et al.* Paradoxical reactions in Buruli ulcer after initiation of antibiotic therapy: Relationship to bacterial load. *PLoS Negl Trop Dis* 2019; **13**: e0007689.

11 Kpeli G, Darko Otchere I, Lamelas A, *et al.* Possible healthcare-associated transmission as a cause of secondary infection and population structure of Staphylococcus aureus isolates from two wound treatment centres in Ghana. *New Microbes New Infect* 2016; **13**: 92–101.

12 Marais S, Cresswell FV, Hamers RL, *et al.* High dose oral rifampicin to improve survival from adult tuberculous meningitis: A randomised placebo-controlled double-blinded phase III trial (the HARVEST study). *Wellcome Open Res* 2020; **4**: 190.

13 Heemskerk AD, Bang ND, Mai NTH, *et al.* Intensified Antituberculosis Therapy in Adults with Tuberculous Meningitis. *N Engl J Med* 2016; **374**: 124–34.

14 Converse PJ, Almeida DV, Tasneen R, *et al.* Shorter-course treatment for Mycobacterium ulcerans disease with high-dose rifamycins and clofazimine in a mouse model of Buruli ulcer. *PLoS Negl Trop Dis* 2018; **12**: e0006728.

15 Totty JP, Bua N, Smith GE, *et al.* Dialkylcarbamoyl chloride (DACC)-coated dressings in the management and prevention of wound infection: a systematic review. *J Wound Care* 2017; **26**: 107–14.

16 Geroult S, Phillips RO, Demangel C. Adhesion of the ulcerative pathogen Mycobacterium ulcerans to DACC-coated dressings. *J Wound Care* 2014; **23**: 417–8, 422–4.

17 iDSI reference case for economic evaluation. iDSI. 2017; published online Aug 29. https://www.idsihealth.org/resource-items/idsi-reference-case-for-economic-evaluation/ (accessed Jan 26, 2019).

18 WHO | Laboratory diagnosis of buruli ulcer. A manual for health care providers. WHO. http://www.who.int/buruli/resources/9789241505703/en/ (accessed Nov 28, 2020).

19 World Health Organization. Buruli Ulcer 01 Reporting Form. .

20 Available EQ-5D versions – EQ-5D. https://euroqol.org/eq-5d-instruments/all-eq-5d-versions/ (accessed Nov 28, 2020).

21 Hunter RM, Baio G, Butt T, Morris S, Round J, Freemantle N. An educational review of the statistical issues in analysing utility data for cost-utility analysis. *PharmacoEconomics* 2015; **33**: 355–66.

22 Sean D. Sullivan, Josephine A. Mauskopf, Federico Augustovski, *et al.,* Budget Impact Analysis—Principles of Good Practice: Report of the ISPOR 2012 Budget Impact Analysis Good Practice II Task Force, Volume 17, Issue 1,2014, Pages 5-14, ISSN 1098-3015

23 Dee A, Hutchinson M, De La Harpe D. A budget impact analysis of natalizumab use in Ireland. Irish Journal of Medical Science 2012 Page 204

24 Wagstaff, A. and Doorslaer, E.v. (2003), Catastrophe and impoverishment in paying for health care: with applications to Vietnam 1993–1998. Health Econ., 12: 921-933. https://doi.org/10.1002/hec.776

25 Adam Wagstaff, Gabriela Flores, Justine Hsu*, et al.,* Progress on catastrophic health spending in 133 countries: a retrospective observational study, The Lancet Global Health, Volume 6, Issue 2, 2018, Pages e169-e179, ISSN 2214-109X, https://doi.org/10.1016/S2214-109X(17)30429-1. (https://www.sciencedirect.com/science/article/pii/S2214109X17304291)
