# Supplementary material for: High-dose rifampicin and clarithromycin for 4 weeks versus standard-dose rifampicin and clarithromycin for 8 weeks to treat Buruli ulcer: An open-label, individually randomized, controlled trial in Ghana
Source: PLoS Negl Trop Dis. 2026 Sep 29;20(9):e0014783. doi: 10.1371/journal.pntd.0014783 (PMC13645219; doi:10.1371/journal.pntd.0014783)
Supplement: Supplementary file 2 [file pntd.0014783.s002.pdf]

S2 SCHEDULES OF PROCEDURES BEFORE, DURING AND AFTER SCREENING

Activity	Screening	Week 0	Week 2	Week 4	Week 6	Week 8	Monthly	12 Monthly	Treatment Failure
Written Informed Consent	**X**								
Inclusion / Exclusion Criteria	**X**	**X**							
Randomisation		**X**							
Socio-demographics	**X**	**X**							
History and Physical Exams		**X**	**X**	**X**	**X**	**X**	**X**	**X**	**X**
Clinical Assessment	**X**	**X**	**X**	**X**	**X**	**X**	**X**	**X**	**X**
Adverse Events			**X**	**X**	**X**	**X**	**X**	**X**	**X**
Clinical Assessment of the Patient and Lesion		**X**	**X**	**X**	**X**	**X**	**X**	**X**	**X**
Measurement of Lesion Size		**X**	**X**	**X**	**X**	**X**	**X**	**X**	**X**
Photograph of lesion by digital camera	**X**	**X**	**X**	**X**	**X**	**X**	**X**	**X**	**X**
Counselling and testing for Pregnancy*	**X**	**X**		**X**		**X**			
Swab/FNA for PCR	**X**	**X**							
Swab/FNA for 16S rRNA (0,2,4,6,8 weeks) and then monthly if not healed		**X**	**X**	**X**	**X**	**X**	**X**		**X**
VAS		**X**	**X**	**X**	**X**	**X**	**X**	**X**	**X**
Functional limitations assessment		**X**	**X**	**X**	**X**	**X**	**X**	**X**	**X**
Health-related quality of life (QALY, DALY) for economic evaluation		**X**	**X**	**X**	**X**	**X**	**X**	**X**	**X**
Household costs of BU and of care seeking		**X**	**X**	**X**	**X**	**X**	**X**	**X**	
Socioeconomic status		**X**	**X**	**X**					
