# Supplementary material for: High-dose rifampicin and clarithromycin for 4 weeks versus standard-dose rifampicin and clarithromycin for 8 weeks to treat Buruli ulcer: An open-label, individually randomized, controlled trial in Ghana
Source: PLoS Negl Trop Dis. 2026 Sep 29;20(9):e0014783. doi: 10.1371/journal.pntd.0014783 (PMC13645219; doi:10.1371/journal.pntd.0014783)
Supplement: Supplementary file 4 [file pntd.0014783.s004.docx]

24^th^ August 2026

Kumasi Centre for Collaborative Research

Kwame Nkrumah University of Science and Technology

Kumasi, Ghana

**The Editor**

**PLoS Neglected Tropical Diseases Journal**

Dear Sir,

**Re: ‘High-dose rifampicin and clarithromycin for 4 weeks versus standard-dose rifampicin and clarithromycin for 8 weeks to treat Buruli ulcer: an open-label, individually randomized, controlled trial in Ghana’ PNTD-D-26-01230**

My co-authors and I have taken note of the review comments and revised the manuscript as suggested. We wish to submit the revised manuscript for publication in your esteemed journal. In the attached reply, the reviewer questions are in black font and our responses are in red font.

We look forward to the next steps towards the publication of our manuscript.

Yours Sincerely,

Yaw Ampem Amoako

**Point-by-point response to review comments**

**Journal Requirements:**

1) Please upload all main figures as separate Figure files in .tif or .eps format. For more information about how to convert and format your figure files please see our guidelines:
   <https://journals.plos.org/plosntds/s/figures>

Response: Done

2) Your current Financial Disclosure states, "The Skin Health Africa Research Programme (SHARP) was funded by the National Institute for Health and Care Research (NIHR) under its Research and Innovation for Global Health Transformation (RIGHT) Programme [Grant Reference Number NIHR200125]. The funders had no role in study design, data collection and analysis, decision to publish, or preparation of the manuscript.MM is supported by an NIHR Research Professorship (NIHR304258). The views expressed are those of the author(s) and not necessarily those of the NIHR or the Department of Health and Social Care.". However, your funding information on the submission form not indicates NIHR304258. Please indicate by return email the full and correct funding information for your study and confirm the order in which funding contributions should appear. Please be sure to indicate whether the funders played any role in the study design, data collection and analysis, decision to publish, or preparation of the manuscript.
   Response: We have sent the returned email as requested. Further, we confirm that the funding statement should read:

‘The Skin Health Africa Research Programme (SHARP) was funded by the National Institute for Health and Care Research (NIHR) under its Research and Innovation for Global Health Transformation (RIGHT) Programme [Grant Reference Number NIHR200125]. The funders had no role in study design, data collection and analysis, decision to publish, or preparation of the manuscript.MM is supported by an NIHR Research Professorship (NIHR304258). The views expressed are those of the author(s) and not necessarily those of the NIHR or the Department of Health and Social Care.’

**Methods**

Reviewer #1:

- Line 150: why were children < 5 years of age excluded from the study?

Response: We have revised the section by adding the following sentences to the section (line 168-173):

‘Children younger than 5 years were excluded primarily for safety reasons, as high-dose rifampicin was not part of the standard treatment regimen at the time of the study. In addition, the distinct pharmacokinetic characteristics and smaller body size of very young children may necessitate age-specific dosing formulations. Furthermore, the assessment of subjective outcomes, such as pain, and the maintenance of reliable follow-up and treatment adherence may be more challenging in this age group.’

- Line 178: was wound diameter the diameter of ulceration alone or did it include the associated area of wound induration around the ulcer?

Response: Wound diameter included the region of induration around ulcers. The has been clarified in the section (line 200-201)
- Lines 195-197: I have some concerns with the definition used for determining secondary infection. A purulent discharge can result from a paradoxical reaction, and wounds are commonly colonised with bacteria. Therefore, a purulent wound discharge and bacteria isolated from a swab does not confirm a secondary infection. Could the authors please explain in more detail how secondary infection was distinguished from a paradoxical reaction and/or wound colonisation?

Response: The section has been revised appropriately and now reads (line 252-257):

‘Secondary infections were evaluated by trained clinicians using a standardized checklist that assessed the presence of pain, erythema, warmth, swelling, and purulent discharge to identify signs suggesting secondary bacterial infection as previously described [27]. A secondary infection was defined as the occurrence of new purulent discharge within a BU lesion in the presence of clinical signs of infection and accompanied by microbiological evidence of infection.’
- Could the authors also please include the definitions used for:
o Recurrence
o Paradoxical reaction

These definitions were provided in the published stauy protocol (ref xx)

Response: In the section on ‘Definition of terms’ these have now been defined (line 237-251).

‘Recurrence was defined as the appearance of a new Buruli ulcer lesion within 12 months of completing antibiotic treatment, after complete healing of the initial lesion, and not considered a paradoxical reaction. This was assessed by the presence of viable micro-organisms detected by *M. ulcerans* culture or the combined *M. ulcerans* IS2404-16S rRNA assay.

Paradoxical reaction was defined as:

a. an initial improvement in the clinical appearance of lesion upon antimicrobial treatment of BU followed by deterioration of the lesion or its surrounding tissues

b. or the appearance of a new lesion(s)

c. or examination of excised tissue from the clinical lesion showing evidence of an intense inflammatory reaction consistent with a paradoxical reaction.’

- Line 206: why was the time to clearance based on time from randomisation, and not from time of antibiotic treatment initiation?

Response: Antibiotic treatment was started on the same day as randomization. This has been clarified in the manuscript text (line 230)

Reviewer #2: In the manuscript “High-dose rifampicin and clarithromycin for 4 weeks versus standard-dose rifampicin and clarithromycin for 8 weeks to treat Buruli ulcer: an open-label, individually randomized, controlled trial in Ghana” the authors report on a trial that was stopped early due to slow recruitment. The fact that these findings are nevertheless written up for publication is commendable.

Major comments:
1. The introduction highlights the features of BU in West Africa yet does not allude to declining incidence estimates based on declining case notifications. This is probably the major reason why recruitment was slower than anticipated, leading to early stopping of the trial. Now it is unclear why recruitment was slow, to the point that the DSMB stopped the trial. Per the supplementary study protocol, which has a different name, the originally foreseen recruitment period was 2,5 years. What proportion of notified cases during the recruitment period were recruited into the trial?

Response: As indicated in the CONSORT diagram, during the recruitment period, 52 Buruli ulcer cases were notified, of which 42 were recruited into the trial, representing a recruitment proportion of 80.8%.
2. Line 228: (mITT) population of individuals who were PCR positive for M. ulcerans… But inclusion criterion was PCR positivity at baseline? Were patients with negative viability assay at baseline included in the mITT analysis?

Response: The modified intention-to-treat (mITT) population included all participants who met the eligibility criteria and were PCR positive for *M. ulcerans* at baseline. Baseline viability assay results were not part of the inclusion criteria and were therefore not used to determine eligibility for the mITT population. Consequently, participants who were PCR positive at baseline but had a negative viability assay result were retained in the mITT analysis.
3. Line 267: No participants in either arm experienced recurrence of BU. Please calculate the CI around 0%. What would the CI have been if full sample size would have been obtained? If relapse free cure is the ultimate objective of treatment of mycobacterial diseases, even if this was not the primary outcome of the trial, what would it take to demonstrate that 4 weeks of high dose rifampicin together with DACC dressings suffices? Please expand on this in the discussion, line 326- what would be the sample size for a non-inferiority trial based on the current findings?

Response: We are not aware of any statistical methods to obtain useful confidence intervals for the odds ratio or risk ratio between two groups where there are zero events in both groups; the best estimate of any confidence interval in this setting is zero to infinity, which is rather uninformative.

We cannot estimate from the information available from this trial what the confidence interval could have been with a larger sample.

**Results**

Reviewer #1:

- Line 258: Table 1: could authors please provide p-values for the comparisons of the reported baseline variables between the 2 arms of the study to allow readers a better understanding of the significance of the comparison of baseline variables between the 2 study groups.

Response: We have decided not to include the p-values comparing baseline characteristics following CONSORT guidelines for the reporting of results of randomized controlled trials which strongly recommend against this practice.
- Line 262: As weight will have an impact on the level of antibiotic in the body, why was weight not adjusted as a co-variable?

Response: The dosing of the administered antibiotics in both arms were already weight adjusted (dose/ kg body weight).
- Line 290: Table 3. I have concerns with the reported adverse effects data which I think is somewhat misleading:
o The study has included urine discoloration as an adverse reaction. This is not an adverse reaction but a normal outcome of taking rifampicin which discolours the urine. I therefore feel this should therefore be removed from the adverse events table.

Response: Reddish-orange discolouration of body fluids such as urine is a well-recognized harmless side effect of rifampicin use and is listed in pharmacopoeia. We have therefore maintained it in the adverse event table.
o The column for SR+DACC reports total adverse events of 15, but then only lists 10 adverse events (3 mucocut/derm, 1 constitutional, 1 anorexia/LOA, and 5 urine discoloration). Thus either there are other adverse reactions that are not reported in the table or in fact there are only 10 instead of 15 (which should be 5 as mentioned above urine discoloration is not an adverse effect).

Response: The table has been revised to address the review concern
o If I am correct then there are 13 AEs in HR group and only 5 in SR group – this suggests there may be a potential for increased toxicity in the HR arm (with larger study numbers) which should be discussed further in the paper.

Response: The table has been revised to address the review concern.

Reviewer #2:

4. Line 271: secondary infections occurred… what was the definition of secondary infections? Were these positive microbial cultures (i.e. colonization) or clinical signs of surrounding soft tissue redness and swelling? How were these managed? Were additional systemic or topical antibiotics used (not per line 275)? What was the timing of secondary infections?

Response: The definition of secondary infection has been included (line 252-257). No additional antibiotics were given for secondary infections. This is reflected in the statement in line 326-327 that;

‘No concomitant medications were prescribed or administered by the study team in either arm during the study’
5. Line 311: The notably lower incidence of paradoxical reactions… The 2025 paper referenced earlier, on high dose rifampicin in meningitis, showed a higher mortality in the high dose rifampicin arm, albeit not significantly higher. One hypothesis could be that faster killing releases more antigen and fuels paradoxical reactions (and hence intracranial pressure).

Response: We are not aware of any clear evidence that clearance of mycobacteria increases risk for paradoxical reactions in BU. Indeed, a previous study from our group showed that the incidence of paradoxical reaction in BU was influenced by the bacterial load (Frimpong M, Agbavor B, Duah MS, Loglo A, Sarpong FN, Boakye-Appiah J, et al. Paradoxical reactions in Buruli ulcer after initiation of antibiotic therapy: Relationship to bacterial load. PLoS Negl Trop Dis. 2019;13(8):e0007689) thus our hypothesis that more rapid bacterial clearance in the high dose arm will likely be associated with fewer PR events.

In the discussion section (line 382-384) we indicate that ‘The marked reduction of paradoxical reactions in the HR arm may be attributed to shorter antigenic stimulation due to more rapid bacterial clearance in the high dose arm.’
6. The main outcome is an RNA based viability marker, yet no results are provided on the detailed results, at baseline nor during treatment, except for the analysis in Table 2. The frequency of measuring is not clear either. Please include a table on (degree of) positivity by timepoint, including the proportion of participants who contributed a sample, and the proportion of samples with sufficient DNA for a robust analysis of RNA presence/ absence.

Response: We have presented these results in Table 3 as requested.

Reviewer #3:

-Does the analysis presented match the analysis plan?
YES
-Are the results clearly and completely presented?
NO - we have no definition of "Paradoxical reaction" or how there were adjudicated; we do not have tabulated results of the viability assay (qPCR) even in a supplementary table
-Are the figures (Tables, Images) of sufficient quality for clarity?
Figures, tables and images addressing paradoxical reactions and the viability assay results would help support the claims the author are making (possible trend towards faster sterilisation, fewer paradoxical reactions) which at the moment we are asked to accept with insufficient detail about how these conclusions were reached.

Response: The results of the viability assays have been included as Table 3.

**Conclusions**
Reviewer #1:

- Line 53: the statement that ‘high dose rifampicin was associated with fewer paradoxical reactions’ is not supported by the data in the trial. In fact, there was no statistical significance in the occurrence of PRs between the two arms as described in line 270. This is again repeated in lines 73-74. I would suggest these statements need to be modified to reflect that no statistically significant difference for PRs was found between the study arms. Furthermore, the discussion in lines 311-315 also needs to be modified to reflect this.

Response: We have modified the sections as suggested.

The section in the abstract now reads (line 53-55):

‘High‑dose rifampicin for 4 weeks was well tolerated in this population. A nominally lower proportion of paradoxical reactions was observed in the high-dose rifampicin arm compared with standard therapy.’

Further, the section in the discussion has been modified as follows (line 363-366):

‘Although paradoxical reactions occurred less frequently in the high-dose rifampicin arm, there was no evidence of a difference in their occurrence between the study arms and recurrence rates were zero in both groups.’
- Based on the concerns with the reporting of adverse effects, the unbalanced randomisation between the 2 groups, and the reduced numbers enrolled in the study compared to the planned enrolment, I would recommend that the conclusions are modified to reflect these uncertainties with study outcomes. For instance for conclusions in line 53: ‘high dose rifampicin may be safe’, in line 55-56 ‘larger trials are need to determine efficacy and safety’, in line 71 ‘but may be safe...’, in line 302 ‘but it may be safe..’ and in line 77 ‘larger studies are need to confirm its efficacy, safety and..’

Response: The sections has been modified as suggested.

Reviewer #2: See above

Reviewer #3: -Are the conclusions supported by the data presented?
NO - we need more detail about the definitions used for paradoxical reactions and the process of adjudication and about the results of the viability assay at least in summary form

Response: The definition of paradoxical reactions has been included (line 246-251). The results of the viability assays have been included as Table 3.

**Editorial and Data Presentation Modifications?**

Reviewer #1:

- The 2 groups compared in this study are not equivalent, despite the randomisation. There are significant differences in age, sex and weight – all of which can have effects on antibiotic toxicity and effectiveness. Therefore, authors need to be very circumspect in their conclusions when comparing outcomes between the 2 groups. I would recommend that this is addressed in the discussion section, but also ensure that conclusions take this into account (for example in line 72 ‘although we enrolled fewer participants than planned and the intervention and control groups were not balanced’.

Response: Done as suggested (line 74)
- Lines 95-98. Additional concerns with current regimens that should be mentioned are high rates of drug toxicity and drug interactions with other medication, especially in older populations.

Response: We have revised the section by adding the following sentences (line 101-103):

‘Additional challenges with the current rifampicin-clarithromycin regimen are the relatively long 8-week duration, adverse effects and drug interactions especially in older populations.’
- I would recommend that there should be some discussion in the paper that there is some evidence to suggest that current regimens, especially rifampicin/clarithromycin combinations, result in suboptimal levels of antibiotics, especially in those with increased weights, that may contribute to treatment failure (O’Brien et al, AAC 2020 Aug 20;64(9):e00722-20). This supports exploration of the use of higher dose rifampicin regimens

Response: We have added text to highlight the potential role of inadequate drug exposure as a factor influencing treatment outcomes by adding the following sentences to the introduction section (line 114-119):

‘Some data suggest that standard rifampicin/clarithromycin-containing regimens may result in suboptimal antibiotic exposures, particularly among patients with higher body weight, potentially contributing to treatment failure [17]. These findings highlight the importance of considering drug exposure as a determinant of treatment response and support further evaluation of dose optimization strategies.’

- Line 117: I am not aware that there is any evidence that accelerating clearance of mycobacteria will reduce the risk of paradoxical reactions (in fact there has been some suggestion in previous studies that it may have the opposite effect and increase the risk). If the authors believe this evidence exists then this should be referenced or at least explained why this may theoretically be the case.

Response: We are not aware of any clear evidence in the past that clearance of mycobacteria increases risk for paradoxical reactions in BU. Indeed, a previous study from our group showed that the incidence of paradoxical reaction in BU was influenced by the bacterial load (Frimpong M, Agbavor B, Duah MS, Loglo A, Sarpong FN, Boakye-Appiah J, et al. Paradoxical reactions in Buruli ulcer after initiation of antibiotic therapy: Relationship to bacterial load. PLoS Negl Trop Dis. 2019;13(8):e0007689) thus our hypothesis that more rapid bacterial clearance in the high dose arm will likely be associated with fewer PR events.

In the discussion section (line 382-384) we indicate that ‘The marked reduction of paradoxical reactions in the HR arm may be attributed to shorter antigenic stimulation due to more rapid bacterial clearance in the high dose arm.’

- Line 299: I note that the study has not found that time to clearance was ‘shorter’ – in fact it was not statistically different (especially when adjusted for co-variables). Thus I would recommend that the statement ‘shorter’ should be modified to reflect this such as changing it to ‘similar’.

Response: Even though the adjustment did not show statistical significance, there was a trend of faster sterilisation in the high dose arm, and a trend of reduced paradoxical reactions in the high dose arm. The section has been revised as suggested (line 360; 363-366).
- Line 309-310: I would suggest that the statement ‘this study provides the first signal that a short-course BU treatment regimen may be possible’ is incorrect and fails to acknowledge previous research. Please see previous evidence published from an Australian context that shows antibiotic regimens shorter than the WHO recommended 8 -week treatment can be effective in curing BU lesions without recurrence (O’Brien et al, Clin Infect Dis. 2020 Apr 15;70(9):1993-1997; Cowan et al, PLoS Negl Trop Dis. 2015. 9(2): e0003503) as well as supportive mouse data (Ji et al, AAC 2007; 51:3737–9; Chauffeur et al PLoS Negl Trop Dis 2016; 10:e0005066). Furthermore, based on this, in Australian guidelines, treatment for only 6 weeks is now recommended as a treatment option for smaller lesions in immunocompetent adults. (Muhi et al, Med J Aust 2025).

Response: The section has been revised as follows (line 373-377):

‘Our findings add to the growing body of evidence suggesting that shorter-course antibiotic treatment regimens for Buruli ulcer may be feasible. Previous studies have reported the effectiveness of treatment durations shorter than the standard 8-week regimen in selected patients [17,28]. Our study provides additional evidence from a high-burden endemic setting that may support future evaluation of shortened treatment strategies.’
- Line 313-315: this statement (patients experiencing a PR…) should be referenced

Response: Done as suggested.
- It should be discussed in this paper that the age of patients in the HR arm is young – therefore may not be applicable to other populations where the average age is higher (eg Australian populations where median ages are near 60 years).

Response: To address the review concern, we have added the following sentence to the discussion section (line 392-395):

‘However, given the younger median age of our high-dose rifampicin cohort, these findings may not be generalizable to older endemic populations - notably those in Australia, where the median age of affected individuals approaches 60 years.’

Reviewer #2: Minor comments:
Line 102 several large-scale TB trials are evaluating this strategy… Please summarize the key findings of high dose rifampicin used for TB treatment.

Response: We have added the following sentence to the introduction section (line 108-112):

‘In a study using high dose rifampin among individuals with tuberculous meningitis, no evidence of survival benefit from high-dose rifampin was observed. However, drug-induced liver injury occurred in 8.0% of the participants in the high-dose group and 4.4% of those in the standard-dose group, but no deaths from drug-induced liver injury occurred [15].’

Line 140: Written informed consent was obtained from all participants, and parental or guardian assent was provided for those aged 13 to 17 years… Please rephrase, presumably parental or guardian consent for all under 18 and assent for those aged 13-17?
Response: The section has been revised as suggested (line 155-156)

Line 150: what was the turn-around-time for IS2404 qPCR?
Response: The median turn-around time for IS2404 qPCR assay was 5 days as previously published [24].’ (line 216-217)

Line 220 … time to clearance of viable mycobacterium with standard dose oral rifampicin… Please provide reference.

Response: Done as requested (line 263)
Table 1: please provide OR and 95% CI for each row.

Response: The CONSORT guidelines for the reporting of results of randomized controlled trials, recommends that this table be purely descriptive. We have opted to follow the CONSORT guidelines and not provide OR and 95% CI
No need to include an interview guide for stigma in supplement- these results are not mentioned.

Response: This has been deleted

**Summary and General Comments**
Reviewer #1: This is an interesting paper that adds to the evidence around the possibility of shortening the duration of antibiotic treatment for Buruli ulcer - something that if achieved would have significant benefits for the safety, feasibility and acceptability of treatment. Therefore, I would recommend that it be published.

However, due to the small numbers, unbalanced randomisation, and questions over some of the data presented, I think that there are some major revisions that need to be considered and that the strength of the conclusions regarding the data presented should be modified. I also think that paper would benefit by the authors strengthening their discussion by broadening their literature review and considering the applicability of their findings to other populations who are affected by BU.

Response: The manuscript has been modified as per all review comments.

Reviewer #2: Please see above

Reviewer #3: This manuscript concisely describes a randomised study of antibiotic therapy performed in Ghana for patients with Buruli ulcer. It compares short course high dose rifampicin with standard dose standard course rifampicin (clarithromycin dose and dressings are the same in each arm). The study had to be stopped early due to slow recruitment, nevertheless the information obtained is valuable and this study is definitely worth publishing. However we need clearer information on which to judge the two major outcomes - similar in the end but possibly a trend towards faster sterilisation in the high dose arm, and a trend towards reduced paradoxical reactions in the high dose arm. To improve data presentation I recommend some form or tabulated results from the viability assays per patient. With respect to paradoxical reactions, while in the methods section definitions of complete healing and secondary infection are provided, there is no definition of paradoxical reaction or information about how paradoxical reaction presence/absence was adjudicated.

Response: The manuscript has been revised as suggested. The definitions of terms and the tabulation of the results of the viability assay have been included as suggested.
