# Supplementary material for: High-dose rifampicin and clarithromycin for 4 weeks versus standard-dose rifampicin and clarithromycin for 8 weeks to treat Buruli ulcer: An open-label, individually randomized, controlled trial in Ghana
Source: PLoS Negl Trop Dis. 2026 Sep 29;20(9):e0014783. doi: 10.1371/journal.pntd.0014783 (PMC13645219; doi:10.1371/journal.pntd.0014783)
Supplement: Peer review history

# Peer review: Decision Letter 0

**DOI:** 10.1371/journal.pntd.0014783.r001

**Authors:** Stuart D. Blacksell, Paul J. Converse

21 Jul 2026

PNTD-D-26-01230

High-dose rifampicin and clarithromycin for 4 weeks versus standard-dose rifampicin and clarithromycin for 8 weeks to treat Buruli ulcer: an open-label, individually randomized, controlled trial in Ghana

PLOS Neglected Tropical Diseases

Dear Dr. Amoako,

Thank you for submitting your manuscript to PLOS Neglected Tropical Diseases. After careful consideration, we feel that it has merit but does not fully meet PLOS Neglected Tropical Diseases's publication criteria as it currently stands. Therefore, we invite you to submit a revised version of the manuscript that addresses the points raised during the review process.

Please submit your revised manuscript within by Sep 19 2026 11:59PM. If you will need more time than this to complete your revisions, please reply to this message or contact the journal office at plosntds@plos.org. When you're ready to submit your revision, log on to https://www.editorialmanager.com/pntd/ and select the 'Submissions Needing Revision' folder to locate your manuscript file.

Please include the following items when submitting your revised manuscript:

* A letter that responds to each point raised by the editor and reviewer(s). You should upload this letter as a separate file labeled 'Response to Reviewers'. This file does not need to include responses to any formatting updates and technical items listed in the 'Journal Requirements' section below.

* A marked-up copy of your manuscript that highlights changes made to the original version. You should upload this as a separate file labeled 'Revised Manuscript with Track Changes'.

* An unmarked version of your revised paper without tracked changes. You should upload this as a separate file labeled 'Manuscript'.

If you would like to make changes to your financial disclosure, competing interests statement, or data availability statement, please make these updates within the submission form at the time of resubmission. Guidelines for resubmitting your figure files are available below the reviewer comments at the end of this letter.

As the corresponding author, your ORCID iD is verified in the submission system and will appear in the published article. PLOS supports the use of ORCID, and we encourage all coauthors to register for an ORCID iD and use it as well. Please encourage your coauthors to verify their ORCID iD within the submission system before final acceptance, as unverified ORCID iDs will not appear in the published article. Only the individual author can complete the verification step; PLOS staff cannot verify ORCID iDs on behalf of authors.

We look forward to receiving your revised manuscript.

Kind regards,

Paul J. Converse

Academic Editor

PLOS Neglected Tropical Diseases

Stuart Blacksell

Section Editor

PLOS Neglected Tropical Diseases

Shaden Kamhawi

co-Editor-in-Chief

PLOS Neglected Tropical Diseases

orcid.org/0000-0003-4304-636XX

Paul Brindley

co-Editor-in-Chief

PLOS Neglected Tropical Diseases

orcid.org/0000-0003-1765-0002

**Additional Editor Comments (if provided):**

Dear Dr. Yaw Ampem Amoako and colleagues,

Your manuscript has reviewed by three experts in the field. Overall they have favorable responses to the paper but all note that there are missing details and definitions for important components that are needed to support your conclusions, notably concerning secondary infections, recuurrence, and paradoxical reactions. Please respond carefully and completely to all concerns in your rebuttal and indicate how you have addressed the concerns and also submit a revised manuscript with changes marked. Thank you for submitting this important work to PLoS Neglected Tropical Diseases.

**Journal Requirements:**

If the reviewer comments include a recommendation to cite specific previously published works, please review and evaluate these publications to determine whether they are relevant and should be cited. There is no requirement to cite these works unless the editor has indicated otherwise.

1) Please upload all main figures as separate Figure files in .tif or .eps format. For more information about how to convert and format your figure files please see our guidelines:

https://journals.plos.org/plosntds/s/figures

2) Your current Financial Disclosure states, "The Skin Health Africa Research Programme (SHARP) was funded by the National Institute for Health and Care Research (NIHR) under its Research and Innovation for Global Health Transformation (RIGHT) Programme [Grant Reference Number NIHR200125]. The funders had no role in study design, data collection and analysis, decision to publish, or preparation of the manuscript.MM is supported by an NIHR Research Professorship (NIHR304258). The views expressed are those of the author(s) and not necessarily those of the NIHR or the Department of Health and Social Care.". However, your funding information on the submission form not indicates NIHR304258. Please indicate by return email the full and correct funding information for your study and confirm the order in which funding contributions should appear. Please be sure to indicate whether the funders played any role in the study design, data collection and analysis, decision to publish, or preparation of the manuscript.

**Reviewers' Comments:**

Reviewer's Responses to Questions

**Key Review Criteria Required for Acceptance?**

As you describe the new analyses required for acceptance, please consider the following:

**Methods**

-Are the objectives of the study clearly articulated with a clear testable hypothesis stated?

-Is the study design appropriate to address the stated objectives?

-Is the population clearly described and appropriate for the hypothesis being tested?

-Is the sample size sufficient to ensure adequate power to address the hypothesis being tested?

-Were correct statistical analysis used to support conclusions?

-Are there concerns about ethical or regulatory requirements being met?

Reviewer #1: - Line 150: why were children < 5 years of age excluded from the study?

- Line 178: was wound diameter the diameter of ulceration alone or did it include the associated area of wound induration around the ulcer?

- Lines 195-197: I have some concerns with the definition used for determining secondary infection. A purulent discharge can result from a paradoxical reaction, and wounds are commonly colonised with bacteria. Therefore, a purulent wound discharge and bacteria isolated from a swab does not confirm a secondary infection. Could the authors please explain in more detail how secondary infection was distinguished from a paradoxical reaction and/or wound colonisation?

- Could the authors also please include the definitions used for:

o Recurrence

o Paradoxical reaction

- Line 206: why was the time to clearance based on time from randomisation, and not from time of antibiotic treatment initiation?

Reviewer #2: In the manuscript “High-dose rifampicin and clarithromycin for 4 weeks versus standard-dose rifampicin and clarithromycin for 8 weeks to treat Buruli ulcer: an open-label, individually randomized, controlled trial in Ghana” the authors report on a trial that was stopped early due to slow recruitment. The fact that these findings are nevertheless written up for publication is commendable.

Major comments:

1. The introduction highlights the features of BU in West Africa yet does not allude to declining incidence estimates based on declining case notifications. This is probably the major reason why recruitment was slower than anticipated, leading to early stopping of the trial. Now it is unclear why recruitment was slow, to the point that the DSMB stopped the trial. Per the supplementary study protocol, which has a different name, the originally foreseen recruitment period was 2,5 years. What proportion of notified cases during the recruitment period were recruited into the trial?

2. Line 228: (mITT) population of individuals who were PCR positive for M. ulcerans… But inclusion criterion was PCR positivity at baseline? Were patients with negative viability assay at baseline included in the mITT analysis?

3. Line 267: No participants in either arm experienced recurrence of BU. Please calculate the CI around 0%. What would the CI have been if full sample size would have been obtained? If relapse free cure is the ultimate objective of treatment of mycobacterial diseases, even if this was not the primary outcome of the trial, what would it take to demonstrate that 4 weeks of high dose rifampicin together with DACC dressings suffices? Please expand on this in the discussion, line 326- what would be the sample size for a non-inferiority trial based on the current findings?

Reviewer #3: -Are the objectives of the study clearly articulated with a clear testable hypothesis stated?

YES

-Is the study design appropriate to address the stated objectives?

YES - stopped early due to slow recruitment but sufficient to show safety and similar efficacy

-Is the population clearly described and appropriate for the hypothesis being tested?

YES

-Is the sample size sufficient to ensure adequate power to address the hypothesis being tested?

NO but this is discussed and justified

-Were correct statistical analysis used to support conclusions?

YES

-Are there concerns about ethical or regulatory requirements being met?

NO

**********

**Results**

-Does the analysis presented match the analysis plan?

-Are the results clearly and completely presented?

-Are the figures (Tables, Images) of sufficient quality for clarity?

Reviewer #1: - Line 258: Table 1: could authors please provide p-values for the comparisons of the reported baseline variables between the 2 arms of the study to allow readers a better understanding of the significance of the comparison of baseline variables between the 2 study groups.

- Line 262: As weight will have an impact on the level of antibiotic in the body, why was weight not adjusted as a co-variable?

- Line 290: Table 3. I have concerns with the reported adverse effects data which I think is somewhat misleading:

o The study has included urine discoloration as an adverse reaction. This is not an adverse reaction but a normal outcome of taking rifampicin which discolours the urine. I therefore feel this should therefore be removed from the adverse events table.

o The column for SR+DACC reports total adverse events of 15, but then only lists 10 adverse events (3 mucocut/derm, 1 constitutional, 1 anorexia/LOA, and 5 urine discoloration). Thus either there are other adverse reactions that are not reported in the table or in fact there are only 10 instead of 15 (which should be 5 as mentioned above urine discoloration is not an adverse effect).

o If I am correct then there are 13 AEs in HR group and only 5 in SR group – this suggests there may be a potential for increased toxicity in the HR arm (with larger study numbers) which should be discussed further in the paper.

Reviewer #2: 4. Line 271: secondary infections occurred… what was the definition of secondary infections? Were these positive microbial cultures (i.e. colonization) or clinical signs of surrounding soft tissue redness and swelling? How were these managed? Were additional systemic or topical antibiotics used (not per line 275)? What was the timing of secondary infections?

5. Line 311: The notably lower incidence of paradoxical reactions… The 2025 paper referenced earlier, on high dose rifampicin in meningitis, showed a higher mortality in the high dose rifampicin arm, albeit not significantly higher. One hypothesis could be that faster killing releases more antigen and fuels paradoxical reactions (and hence intracranial pressure).

6. The main outcome is an RNA based viability marker, yet no results are provided on the detailed results, at baseline nor during treatment, except for the analysis in Table 2. The frequency of measuring is not clear either. Please include a table on (degree of) positivity by timepoint, including the proportion of participants who contributed a sample, and the proportion of samples with sufficient DNA for a robust analysis of RNA presence/ absence.

Reviewer #3: -Does the analysis presented match the analysis plan?

YES

-Are the results clearly and completely presented?

NO - we have no definition of "Paradoxical reaction" or how there were adjudicated; we do not have tabulated results of the viability assay (qPCR) even in a supplementary table

-Are the figures (Tables, Images) of sufficient quality for clarity?

Figures, tables and images addressing paradoxical reactions and the viability assay results would help support the claims the author are making (possible trend towards faster sterilisation, fewer paradoxical reactions) which at the moment we are asked to accept with insufficient detail about how these conclusions were reached.

**********

**Conclusions**

-Are the conclusions supported by the data presented?

-Are the limitations of analysis clearly described?

-Do the authors discuss how these data can be helpful to advance our understanding of the topic under study?

-Is public health relevance addressed?

Reviewer #1: - Line 53: the statement that ‘high dose rifampicin was associated with fewer paradoxical reactions’ is not supported by the data in the trial. In fact, there was no statistical significance in the occurrence of PRs between the two arms as described in line 270. This is again repeated in lines 73-74. I would suggest these statements need to be modified to reflect that no statistically significant difference for PRs was found between the study arms. Furthermore, the discussion in lines 311-315 also needs to be modified to reflect this.

- Based on the concerns with the reporting of adverse effects, the unbalanced randomisation between the 2 groups, and the reduced numbers enrolled in the study compared to the planned enrolment, I would recommend that the conclusions are modified to reflect these uncertainties with study outcomes. For instance for conclusions in line 53: ‘high dose rifampicin may be safe’, in line 55-56 ‘larger trials are need to determine efficacy and safety’, in line 71 ‘but may be safe...’, in line 302 ‘but it may be safe..’ and in line 77 ‘larger studies are need to confirm its efficacy, safety and..’

Reviewer #2: See above

Reviewer #3: -Are the conclusions supported by the data presented?

NO - we need more detail about the definitions used for paradoxical reactions and the process of adjudication and about the results of the viability assay at least in summary form

-Are the limitations of analysis clearly described?

YES

-Do the authors discuss how these data can be helpful to advance our understanding of the topic under study?

YES

-Is public health relevance addressed?

YES

**********

**Editorial and Data Presentation Modifications?**

Use this section for editorial suggestions as well as relatively minor modifications of existing data that would enhance clarity. If the only modifications needed are minor and/or editorial, you may wish to recommend “Minor Revision” or “Accept”.

Reviewer #1: - The 2 groups compared in this study are not equivalent, despite the randomisation. There are significant differences in age, sex and weight – all of which can have effects on antibiotic toxicity and effectiveness. Therefore, authors need to be very circumspect in their conclusions when comparing outcomes between the 2 groups. I would recommend that this is addressed in the discussion section, but also ensure that conclusions take this into account (for example in line 72 ‘although we enrolled fewer participants than planned and the intervention and control groups were not balanced’.

- Lines 95-98. Additional concerns with current regimens that should be mentioned are high rates of drug toxicity and drug interactions with other medication, especially in older populations.

- I would recommend that there should be some discussion in the paper that there is some evidence to suggest that current regimens, especially rifampicin/clarithromycin combinations, result in suboptimal levels of antibiotics, especially in those with increased weights, that may contribute to treatment failure (O’Brien et al, AAC 2020 Aug 20;64(9):e00722-20). This supports exploration of the use of higher dose rifampicin regimens

- Line 117: I am not aware that there is any evidence that accelerating clearance of mycobacteria will reduce the risk of paradoxical reactions (in fact there has been some suggestion in previous studies that it may have the opposite effect and increase the risk). If the authors believe this evidence exists then this should be referenced or at least explained why this may theoretically be the case.

- Line 299: I note that the study has not found that time to clearance was ‘shorter’ – in fact it was not statistically different (especially when adjusted for co-variables). Thus I would recommend that the statement ‘shorter’ should be modified to reflect this such as changing it to ‘similar’.

- Line 309-310: I would suggest that the statement ‘this study provides the first signal that a short-course BU treatment regimen may be possible’ is incorrect and fails to acknowledge previous research. Please see previous evidence published from an Australian context that shows antibiotic regimens shorter than the WHO recommended 8 -week treatment can be effective in curing BU lesions without recurrence (O’Brien et al, Clin Infect Dis. 2020 Apr 15;70(9):1993-1997; Cowan et al, PLoS Negl Trop Dis. 2015. 9(2): e0003503) as well as supportive mouse data (Ji et al, AAC 2007; 51:3737–9; Chauffeur et al PLoS Negl Trop Dis 2016; 10:e0005066). Furthermore, based on this, in Australian guidelines, treatment for only 6 weeks is now recommended as a treatment option for smaller lesions in immunocompetent adults. (Muhi et al, Med J Aust 2025).

- Line 313-315: this statement (patients experiencing a PR…) should be referenced

- It should be discussed in this paper that the age of patients in the HR arm is young – therefore may not be applicable to other populations where the average age is higher (eg Australian populations where median ages are near 60 years).

Reviewer #2: Minor comments:

Line 102 several large-scale TB trials are evaluating this strategy… Please summarize the key findings of high dose rifampicin used for TB treatment.

Line 140: Written informed consent was obtained from all participants, and parental or guardian assent was provided for those aged 13 to 17 years… Please rephrase, presumably parental or guardian consent for all under 18 and assent for those aged 13-17?

Line 150: what was the turn-around-time for IS2404 qPCR?

Line 220 … time to clearance of viable mycobacterium with standard dose oral rifampicin… Please provide reference.

Table 1: please provide OR and 95% CI for each row.

No need to include an interview guide for stigma in supplement- these results are not mentioned.

Reviewer #3: (No Response)

**********

**Summary and General Comments**

Use this section to provide overall comments, discuss strengths/weaknesses of the study, novelty, significance, general execution and scholarship. You may also include additional comments for the author, including concerns about dual publication, research ethics, or publication ethics. If requesting major revision, please articulate the new experiments that are needed.

Reviewer #1: This is an interesting paper that adds to the evidence around the possibility of shortening the duration of antibiotic treatment for Buruli ulcer - something that if achieved would have significant benefits for the safety, feasibility and acceptability of treatment. Therefore, I would recommend that it be published.

However, due to the small numbers, unbalanced randomisation, and questions over some of the data presented, I think that there are some major revisions that need to be considered and that the strength of the conclusions regarding the data presented should be modified. I also think that paper would benefit by the authors strengthening their discussion by broadening their literature review and considering the applicability of their findings to other populations who are affected by BU.

Reviewer #2: Please see above

Reviewer #3: This manuscript concisely describes a randomised study of antibiotic therapy performed in Ghana for patients with Buruli ulcer. It compares short course high dose rifampicin with standard dose standard course rifampicin (clarithromycin dose and dressings are the same in each arm). The study had to be stopped early due to slow recruitment, nevertheless the information obtained is valuable and this study is definitely worth publishing. However we need clearer information on which to judge the two major outcomes - similar in the end but possibly a trend towards faster sterilisation in the high dose arm, and a trend towards reduced paradoxical reactions in the high dose arm. To improve data presentation I recommend some form or tabulated results from the viability assays per patient. With respect to paradoxical reactions, while in the methods section definitions of complete healing and secondary infection are provided, there is no definition of paradoxical reaction or information about how paradoxical reaction presence/absence was adjudicated.

**********

PLOS authors have the option to publish the peer review history of their article (what does this mean?). If published, this will include your full peer review and any attached files.

If you choose “no”, your identity will remain anonymous but your review may still be made public.

**Do you want your identity to be public for this peer review?**  For information about this choice, including consent withdrawal, please see our Privacy Policy.

Reviewer #1: No

Reviewer #2: **Yes:**  Bouke C. de Jong

Reviewer #3: No

[NOTE: If reviewer comments were submitted as an attachment file, they will be attached to this email and accessible via the submission site. Please log into your account, locate the manuscript record, and check for the action link "View Attachments". If this link does not appear, there are no attachment files.]

**Figure resubmission:**

**Reproducibility:**

To enhance the reproducibility of your results, we recommend that authors of applicable studies deposit laboratory protocols in protocols.io, where a protocol can be assigned its own identifier (DOI) such that it can be cited independently in the future. Additionally, PLOS ONE offers an option to publish peer-reviewed clinical study protocols. Read more information on sharing protocols at https://plos.org/protocols?utm_medium=editorial-email&utm_source=authorletters&utm_campaign=protocols

# Author comment: Author response to Decision Letter 1

**DOI:** 10.1371/journal.pntd.0014783.r002

26 Aug 2026

Attachment Submitted filename: Response to Reviewers_23082026.docx

# Editor report: Decision Letter 1

**DOI:** 10.1371/journal.pntd.0014783.r003

**Authors:** Stuart D. Blacksell, Paul J. Converse

4 Sep 2026

Dear Amoako,

We are pleased to inform you that your manuscript 'High-dose rifampicin and clarithromycin for 4 weeks versus standard-dose rifampicin and clarithromycin for 8 weeks to treat Buruli ulcer: an open-label, individually randomized, controlled trial in Ghana' has been provisionally accepted for publication in PLOS Neglected Tropical Diseases.

Before your manuscript can be formally accepted you will need to complete some formatting changes, which you will receive in a follow up email. A member of our team will be in touch with a set of requests.

Please note that your manuscript will not be scheduled for publication until you have made the required changes, so a swift response is appreciated.

IMPORTANT: The editorial review process is now complete. PLOS will only permit corrections to spelling, formatting or significant scientific errors from this point onwards. Requests for major changes, or any which affect the scientific understanding of your work, will cause delays to the publication date of your manuscript.

Should you, your institution's press office or the journal office choose to press release your paper, you will automatically be opted out of early publication. We ask that you notify us now if you or your institution is planning to press release the article. All press must be co-ordinated with PLOS.

Thank you again for supporting Open Access publishing; we are looking forward to publishing your work in PLOS Neglected Tropical Diseases.

Best regards,

Paul J. Converse

Academic Editor

PLOS Neglected Tropical Diseases

Joseph Vinetz

Section Editor

PLOS Neglected Tropical Diseases

Shaden Kamhawi

co-Editor-in-Chief

PLOS Neglected Tropical Diseases

orcid.org/0000-0003-4304-636XX

Paul Brindley

co-Editor-in-Chief

PLOS Neglected Tropical Diseases

orcid.org/0000-0003-1765-0002

***********************************************************

Dear Dr. Yaw Ampem Amoako and colleagues,

Thank you for your careful revision of the manuscript and addressing of the concerns of the reviewers.

# Editor report: Acceptance letter

**DOI:** 10.1371/journal.pntd.0014783.r004

**Authors:** Stuart D. Blacksell, Paul J. Converse

Dear Amoako,

We are delighted to inform you that your manuscript, "High-dose rifampicin and clarithromycin for 4 weeks versus standard-dose rifampicin and clarithromycin for 8 weeks to treat Buruli ulcer: an open-label, individually randomized, controlled trial in Ghana," has been formally accepted for publication in PLOS Neglected Tropical Diseases.

We have now passed your article onto the PLOS Production Department who will complete the rest of the publication process. All authors will receive a confirmation email upon publication.

The corresponding author will soon be receiving a typeset proof for review, to ensure errors have not been introduced during production. Please review the PDF proof of your manuscript carefully, as this is the last chance to correct any scientific or type-setting errors. Please note that major changes, or those which affect the scientific understanding of the work, will likely cause delays to the publication date of your manuscript. Note: Proofs for Front Matter articles (Editorial, Viewpoint, Symposium, Review, etc...) are generated on a different schedule and may not be made available as quickly.

Soon after your final files are uploaded, the early version of your manuscript will be published online unless you opted out of this process. The date of the early version will be your article's publication date. The final article will be published to the same URL, and all versions of the paper will be accessible to readers.

For Research Articles, you will receive an invoice from PLOS for your publication fee after your manuscript has reached the completed accept phase. If you receive an email requesting payment before acceptance or for any other service, this may be a phishing scheme. Learn how to identify phishing emails and protect your accounts at https://explore.plos.org/phishing.

Thank you again for supporting open-access publishing; we are looking forward to publishing your work in PLOS Neglected Tropical Diseases.

Best regards,

Shaden Kamhawi

co-Editor-in-Chief

PLOS Neglected Tropical Diseases

Paul Brindley

co-Editor-in-Chief

PLOS Neglected Tropical Diseases
